# Multiscale modeling of tumor growth and angiogenesis: Evaluation of tumor-targeted therapy

**DOI:** 10.1371/journal.pcbi.1009081

**Published:** 2021-06-23

**Authors:** Sahar Jafari Nivlouei, M. Soltani, João Carvalho, Rui Travasso, Mohammad Reza Salimpour, Ebrahim Shirani

**Affiliations:** 1 Department of Mechanical Engineering, Isfahan University of Technology, Isafahan, Iran; 2 CFisUC, Department of Physics, University of Coimbra, Coimbra, Portugal; 3 Department of Mechanical Engineering, K. N. Toosi University of Technology, Tehran, Iran; 4 Department of Electrical and Computer Engineering, University of Waterloo, Ontario, Canada; 5 Centre for Biotechnology and Bioengineering (CBB), University of Waterloo, Waterloo, Ontario, Canada; 6 Advanced Bioengineering Initiative Center, Computational Medicine Center, K. N. Toosi University of Technology, Tehran, Iran; 7 Cancer Biology Research Center, Cancer Institute of Iran, Tehran University of Medical Sciences, Tehran, Iran; 8 Department of Mechanical Engineering, Foolad Institute of Technology, Fooladshahr, Iran; Oxford, UNITED KINGDOM

## Abstract

The dynamics of tumor growth and associated events cover multiple time and spatial scales, generally including extracellular, cellular and intracellular modifications. The main goal of this study is to model the biological and physical behavior of tumor evolution in presence of normal healthy tissue, considering a variety of events involved in the process. These include hyper and hypoactivation of signaling pathways during tumor growth, vessels’ growth, intratumoral vascularization and competition of cancer cells with healthy host tissue. The work addresses two distinctive phases in tumor development—the avascular and vascular phases—and in each stage two cases are considered—with and without normal healthy cells. The tumor growth rate increases considerably as closed vessel loops (anastomoses) form around the tumor cells resulting from tumor induced vascularization. When taking into account the host tissue around the tumor, the results show that competition between normal cells and cancer cells leads to the formation of a hypoxic tumor core within a relatively short period of time. Moreover, a dense intratumoral vascular network is formed throughout the entire lesion as a sign of a high malignancy grade, which is consistent with reported experimental data for several types of solid carcinomas. In comparison with other mathematical models of tumor development, in this work we introduce a multiscale simulation that models the cellular interactions and cell behavior as a consequence of the activation of oncogenes and deactivation of gene signaling pathways within each cell. Simulating a therapy that blocks relevant signaling pathways results in the prevention of further tumor growth and leads to an expressive decrease in its size (82% in the simulation).

## Author summary

Mathematical modeling and simulation of cancer across different biological scales is becoming increasingly important in the development of therapeutic strategies. In the current work, a multiscale model is presented to study the growth and progression of tumor and angiogenesis based on tumor-host interactions which allows investigating the effects of tumor-targeted therapy. Considering the signal-transduction networks involved in various types of cancers, we proposed a cascade that encompasses some significant signaling pathways. A Boolean network model is employed to describe the receptors cross-talk. As a result of the activation of oncogenes and deactivation of pertinent gene signaling pathways within each cell, the cellular interactions and cell behavior are modeled. By linking cells state with environmental cues, the tumor morphology is determined. Consistent with the experimental observations, the intratumoral vascularization density resulting from the simulation reports malignancy grade as a prognostic parameter. Moreover, our model permits to explore possible novel therapeutic procedures, including therapies targeting specific pathways. It captures cellular apoptosis by receptor inhibition in tumor development as a new area of mathematical modeling of targeted therapy.

## Introduction

**Fig 1:**
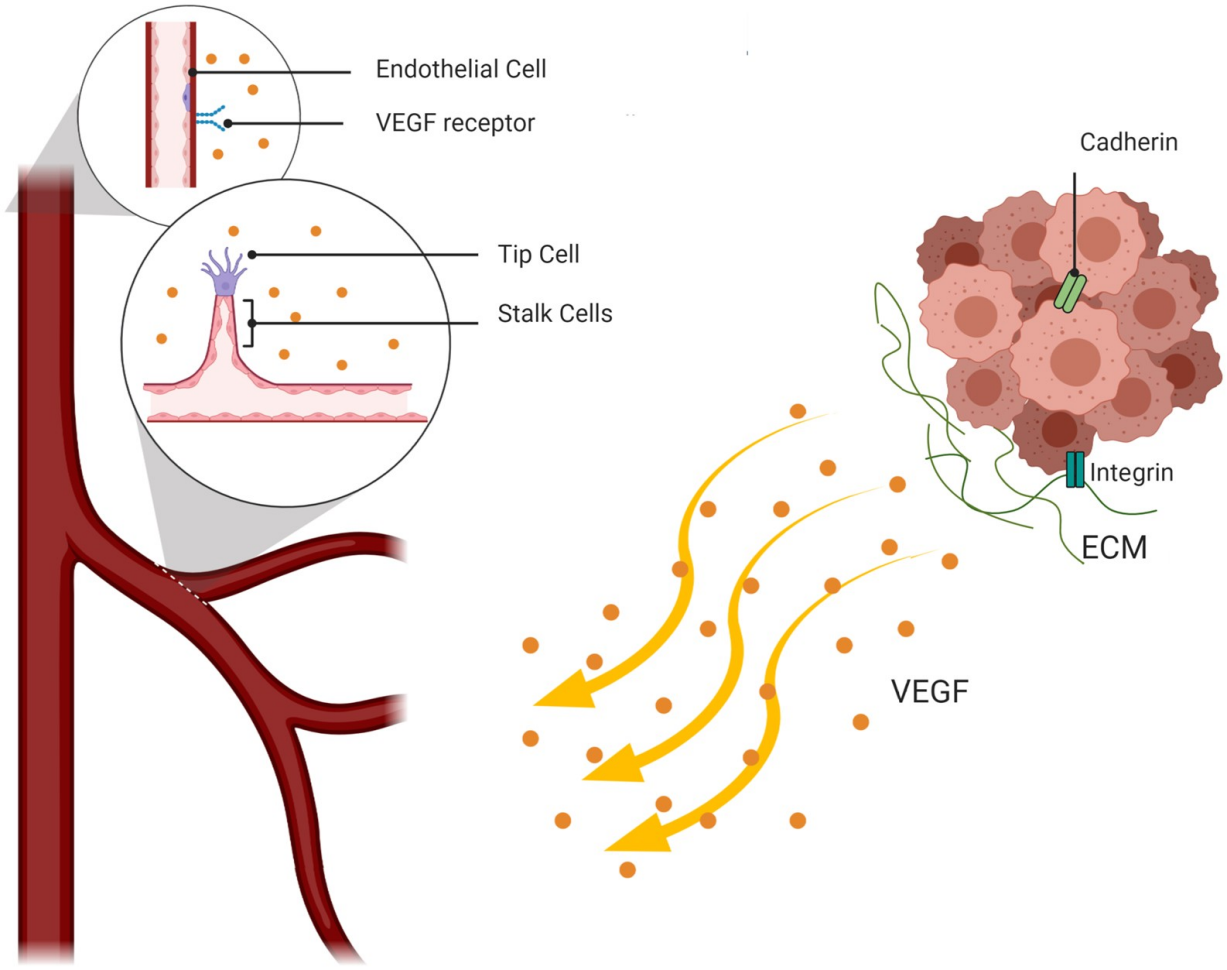
A diagram identifying important events in angiogenesis at the cellular level. Tumor cells secrete VEGF that drives the activation of Endothelial cells by binding to VEGF-receptors on the ECs’ surface. VEGF meditated ECs undertake two distinct phenotypes: tip and stalk cell. Tip cells migrate towards the tumor to reach the VEGF source and highly proliferative stalk cells form the new sprouts body.

Tumor development leads to an accumulation of abnormal cells with high capacity to proliferate and resist apoptosis. The uncontrollable cell proliferation behavior is caused by genetic and epigenetic changes, which lead to the deregulation of cell signaling pathways that maintain normal cellular function. Initially, solid tumors grow in an avascular mode. Due to the limitation of nutrient diffusion from surrounding vessels, the cells in the tumor core suffer hypoxia. In order to promote cell survival in response to this new condition, cells express hypoxia-inducible factor-1 (HIF-1) which up-regulates pro-angiogenic factors, including the vascular endothelial growth factor (VEGF), triggering tumor vascularization (Fig 1).

Tumors in the vascular growth phase are more likely to be aggressive since they have easier access to nearby blood vessels, and thus the metastasis process is facilitated. However, tumor metastatic progression often occurs during the avascular growth in already highly vascularized organs such as brain, lung, liver and lymph nodes [1].

The nature of tumor growth and related events cover multicellular dynamics in different spatio-temporal scales, generally including tissue and extracellular, cellular and intracellular processes. However, mathematical models of the processes relevant to tumor development and angiogenesis often cover only a limited set of events at a specific scale. Continuous models are based on standard advection-diffusion-reaction equations that describe the dynamics of the process at tissue scale, without a detailed description at cellular level [2–4]. In contrast, discrete models, such as cellular automata and agent-based models, handle interactions by considering cells as distinct components. Although results are biologically guided, it is computationally expensive [5,6]. Hence hybrid stochastic models are the most suitable modeling technique because of their ability to span multiple scales [7–11].

Anderson [12] developed a hybrid mathematical model of the solid tumor invasion process. In this study, tumor cells are discrete agents and their behavior is based on a biased random-walk model, while the extracellular matrix, matrix-degrading enzymes and oxygen are modeled as continuous variables [12]. In this work it has been assumed that extracellular matrix is the host tissue. The model discusses the effect of cell interactions on the final structure of tumor due to reducing the oxygen concentration in a homogeneous ECM. Indeed, oxygen availability determines the tumor shape.

The mathematical models of tumor induced microvasculature have also been substantially explored over the past few decades to describe tumor-induced angiogenesis and blood flow in a vascular network [13–15]. See [16–19] for reviews of mathematical models of angiogenesis. In the context of hybrid models of angiogenesis, Travasso et al. [17] developed a multi-scale phase-field model that includes a cellular automaton for the vessel tip cells to track the interface between newly formed vascular networks and the microenvironment. The network morphology is determined by tip cell migration velocity while the stalk cell proliferation helps vascular expansion. They demonstrated how higher tip cell velocity leads to thinner vessels in the vasculature network. However, the matrix rigidity is neglected and anastomosis formation was not taken into account. Moreira-Soares et al. [10] improved the model presented in [17] that simulates the ECs and the ECM using the phase-field approach. They studied of vessel growth and investigated the role of angiogenic factor gradients produced by hypoxic cells, in anastomosis formation. The phase-field technique was also used in a continuous model by Santos-Oliveira et al. [20] to model sprouting angiogenesis as a function of the cell-cell adhesion and the traction forces. This continuous model was more complete as the proliferation of the sprout stalk cells is triggered by both VEGF and the traction forces exerted by the tip cell. It has been reported that higher traction and adhesion forces lead to sprout breaking. Continuous models also help to explore blood flow through a capillary network [21–23]. Soltani and Chen [22] presented a continuous model that investigated interstitial fluid flow patterns in the capillaries and tumor’s surrounding tissue by the solution of Navier–Stokes equations. The model calculates the interstitial pressure as a barrier to drug transport and also has been developed to investigate tumors topological changes and its effects on drug delivery [23–25]. However, continuous models do not provide cells dynamics based on cellular interactions, and then makes it less suitable to understand the individual cells’ role during tumor growth and angiogenesis.

More recently, multiscale models investigated the cellular and molecular mechanisms to reproduce the real biological and physical mechanisms of tumor growth and angiogenesis [19,26–29]. Owen et al. [30] developed a multiscale model that considers subcellular scales built on the study of Alarcón et al. [31–33]. They studied the effect of blood flow and vascular remodeling during angiogenesis and combined tissue scale and the subcellular dynamics of multiple cell populations. To investigate tumor’s development dynamics, Perfahl et al. [34] extended the model proposed by Owen et al. [30] into a 3D multiscale model of vascular tumor growth, including nutrient/growth factor transport and interactions between normal and tumor cells. The authors predict the patterns of vascular remodeling. Similarly, a hybrid CA model of tumor growth within a digitized vasculature was addressed by Stephanou et al. [35,36]. They reported tumor dormancy as a potential consequence of intense vascular changes in the host tissue. A less efficient vascular network leads to less oxygen delivery to tumor cells, which changes cell proliferative behavior to the hypoxic state.

Although the discussed hybrid models have a good potential to predict tumor evolution, all lack description of cell dynamics based on cell mechanical forces. Cellular Potts Models (CPM) of tumor growth and angiogenesis have been successful in dynamically capturing cell shape changes, determining the structure of vasculature, and tracking the interactions between cells and ECM [8,11,26,27,37]. Bauer et al. [26,27] presented a CPM model of sprouting angiogenesis to investigate extracellular matrix (ECM) structure influence in this context. The authors studied the phenotype changes of endothelial cells, induced by the signaling pathways, using a Boolean network model. They demonstrated that sprout migration and structure is influenced by anisotropies in the stroma that plays a key role in regulating branching and anastomosis. Bazmara et al. [38] used these results and developed a model that can predict the formation of a closed blood flow loop (anastomosis), driven by shear stress activation of ECs cells. An alternative study by Vega and co-workers presented a model of early stage angiogenesis in which tip cell selection and vessel branching are determined by Jagged-Notch and Delta-Notch dynamics [39]. The model was more complete and investigates the branching of blood vessels, anastomosis and angiogenesis velocity. Shirinifard et al. [8] presented a 3D multi-scale CPM model which simulates tumor avascular growth and the transition to the vascular phase. This model takes into account the local oxygen levels and how they determine the cells’ different responses.

In this paper, a multiscale model of 2D tumor vascular growth is developed to couple multiple time and length scales. The model considers the mechanical interactions between cancer cells, healthy tissue, adjacent endothelial cells and the ECM, as a consequence of the relevant biochemical mechanisms, which makes it a relevant and novel to mathematical model of tumor growth. Hence, three scale levels: intracellular, cellular, and tissue are taken into consideration. To construct a realistic model, the changes of system behavior are determined by the cells’ signaling pathways, at the intracellular scale. A Boolean network model is used to express the relation between input signals and cross talk between receptors. At the cellular scale, the interaction of different types of cells between themselves and with the micro-environment is modeled using a cell-based cellular Potts model. In addition, normal cells are considered as the host tissue. At the tissue scale, the production of a new vascular network around the tumor and the dynamics of tissue topology are modeled. Diffusion, uptake, and decay of vessel-secreted nutrients and the tumor-secreted VEGF are described through a set of partial differential equations. All the scales involved are integrated simultaneously to study the system dynamics. The model surveys the external environment role on the state of each cell, which can be of proliferation, migration, or apoptosis. Response to external cues determines the tumor growth morphology. The model couples the relevant pathways in each cell and determines the state of cell by linking it with its environmental cues. Moreover, it helps to investigate cancer treatment strategies and mechanisms of controlling disease’s progression. In particular, the model permits to explore the consequence of targeted receptor inhibition in tumor development.

## Material and methods

The multiscale model of the current work covers intracellular, cellular, and tissue scales. The description of each scale is presented in a separate section, as follows.

### Signal transduction—Intracellular level

Cancer initiation and development is associated with mutations that lead to activation of proliferative, anti-apoptotic, and migratory signaling pathways. While tumor promotion depends on the activation of growth factors signals, such as RTKs, the Raf-MEK-ERK mitogen-activated protein kinase, anti-apoptotic pathways are a feature of tumor cell survival. Activity of anti-apoptotic pathways relies on decreasing the expression of tumor suppressor genes such as NF1 and APC [40,41]. When the tumor becomes more aggressive and cancer metastasis occurs, the main members of Rho GTPase family, the RhoA-related and the Rac1-related subfamilies, are up-regulated [42,43]. Metastases are mainly driven by cell motility that triggers morphological changes in the tumor by modifications in actin polymerization, which drives cytoskeleton rearrangements [44]. The full range of events involved in tumor development are affected by continuous interactions with the extracellular matrix (ECM) components and alterations in signaling pathways that regulate cell adhesion [45]. Integrins are cell surface receptors of various ECM proteins involved in cell-adhesion and drive cells binding to the ECM. The expression of these transmembrane receptors is affected in the metastatic process, as they regulate various cellular functions and are important determinants of cell viability.

Cell survival in multicellular organisms not only depends on the availability of nutrients in the surrounding tissue, but also on the continuous interaction with adjoining cells, as cell proliferation is regulated through the contact between cells [46]. The cell–cell adhesion receptor, E-cadherin, has a crucial role in tumor progression, and its loss of function promotes cancer. Generally, E-cadherin facilitates the interaction between cells and keeps them together [47–49]. Indeed, it mediates contact inhibition of cell growth and proliferation. In a healthy tissue, the adequate level of E-cadherin homophilic binding between cells controls the proliferation and forms the adherent junctions [50,51]. Therefore, loss of E-cadherin expression leads to loss of contact inhibition of proliferation and allows cells to grow on top of each other.

Furthermore, cadherin plays an important role in formation of new vessels and expansion of microvascular networks. The VE-cadherin, a transmembrane receptor specific of endothelial cells, is known as the main adherent junction protein and it is indispensable for angiogenesis in response to VEGF stimulation [52]. During sprouting angiogenesis, endothelial cells move through chemotaxis along the VEGF gradient. Two distinct phenotypes of the endothelial cells develop, namely the tip and the stalk cell phenotypes [53]. Tip cells have a motile behavior, in comparison with stalk cells, which proliferate behind the tip cell and build the lumen. The role of VE-cadherin is clear in the regulation of contact inhibition of stalk cell proliferation, and controls cell-cell adhesion junctions to form new functional vessels [54].

It should be noted that the cadherin-catenin is an important aspect in the regulation of cell proliferation and invasion during cancer development and progression. E-cadherin receptor is associated with ß-catenin and binds to the cell’s cytoskeleton that mediates downstream signaling pathways, including the Wnt [55–58]. ß-catenin is the main effector of the Wnt signaling pathway, which is stabilized by activation of Wnt and translocates to the nucleus [59,60]. In addition, apart from Wnt signaling pathway, it has a key role as it mediates the interaction of cadherin with the actin cytoskeleton. Experimental reports demonstrate that reduction in cadherin levels enhances nuclear ß-catenin by releasing it at the cell surface, stimulating Wnt signaling [61]. Indeed, the loss of E-cadherin expression leads to accumulation of membrane-bound ß-catenin, which means that the ß-catenin signaling through the Wnt pathway is negatively regulated by E-cadherin. Interestingly, re-expression of E-cadherin in cells prevents activation of downstream signals and acts as a barrier to tumor progression, since it blocks cell movement.

**Fig 2:**
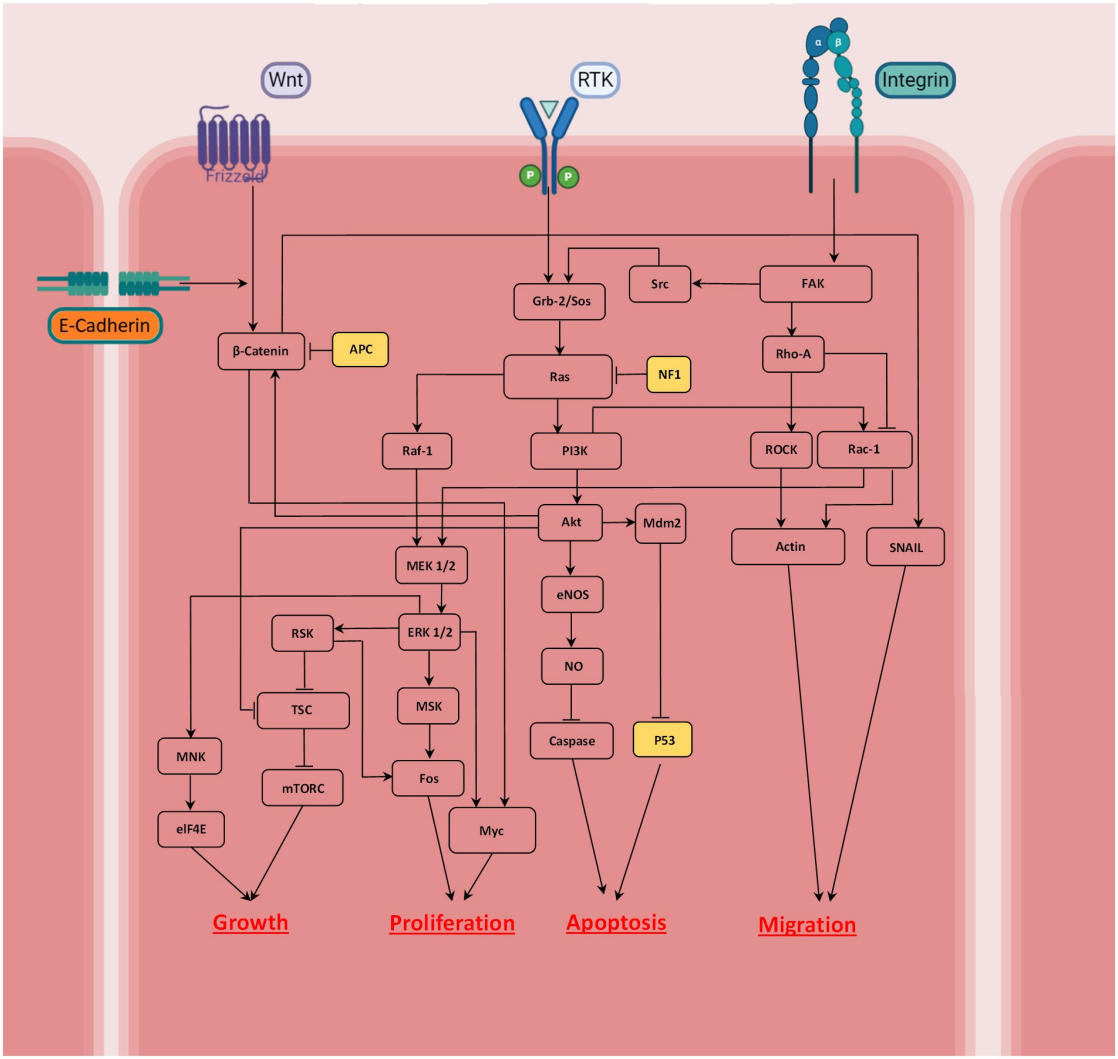
Signaling networks considered in the model that are involved in tumor growth; the implemented network focuses on Ras- PI3K-Akt and Wnt/ß-catenin signaling that control the cancer cell state. Highlighting the relationship between external stimuli, Wnt, RTK, cadherin and integrin, the cell can be growing, proliferating, migrating or undergoing apoptosis. A pointed head arrow indicates activation whereas a blunt head refers to inhibition.

Considering the main receptors involved in tumor development, the model builds a signaling cascade highlighting the cross-talk between growth factors (RTKs), integrin, cadherin and Wnt. Multiple observations are integrated in the proposed signaling network, as shown in Fig 2. The activation or inhibition of each effector in the network is considered as Boolean dependent relations. Pointed head arrows designate activation of each effector while blunt head lines indicate inhibition. The dependence relations are given separately by the corresponding references in Table 1.

**Table 1:** Boolean dependence relations between molecules of the signaling cascades presented in Fig 2, and the corresponding references.

Node	Dependence Relation	Reference
Integrin	External signal (Integrin binding)	[45]
RTK	External signal (VEGF binding)	[26,27]
E-Cadherin	External signal (cadherin binding)	[47–49]
Wnt	External signal (Frizzled receptor)	[55–58]
ß-Catenin	Wnt Or Akt And Not cadherin AND Not APC	[59,60]
APC	External signal	[40]
Grb-2/Sos	RTK And Scr	[62]
Src	FAK	[63,64]
FAK	ITG	[64]
Rho-A	FAK	[64]
ROCK	Rho-A	[65,66]
Rac-1	PI3K And Not Rho-A	[42,43]
Ras	Grb-2/Sos And Not NF1	[62,67,68]
NF1	External signal	[41]
Raf-1	Ras	[69–71]
MEK1/2	Raf-1 Or Rac-1	[69,72]
ERK1/2	MEK1/2	[69,72]
RSK	ERK 1/2	[73,74]
TSC	Not RSK Or Not Akt	[74,75]
mTORC	Not TSC	[76]
MNK	ERK 1/2	[77]
eIF4E	MNK	[78]
MSK	ERK 1/2	[79]
Fos	MSK And RSK	[80,81]
Myc	ERK 1/2 Or ß-Catenin	[82,83]
PI3K	Ras	[69]
Akt	PI3K	[84,85]
eNOS	Akt	[86]
NO	eNOS	[87]
Caspase	Not NO	[85]
Mdm2	Akt	[88]
p53	Not Mdm2	[89]
Actin	ROCK Or Rac-1	[43]
SNAIL	ß-Catenin	[90]
Cell growth	eIF4E Or mTORC	[76,78]
Cell Proliferation	Fos And Myc	[69,73,91]
Cell Apoptosis	Caspase Or p53	[88,92]
Cell Migration	Actin And SNAIL	[43,90]

As the quantitative information on the kinetics of the biochemical reactions of signal transduction pathways is scarce, a Boolean network model is employed to model the signaling cascade regulation and the relation between input signals and cross talk between effective receptors. Boolean networks are a helpful tool to model dynamics of gene regulatory networks, as introduced by Stuart Kauffman [93,94]. Accordingly, Bauer and co-workers [95] constructed a Boolean network model of receptor cross-talk involved in angiogenesis to suggest molecular targets for anti-angiogenic therapies. Boolean network analysis provides the dynamic behavior of the signaling network in the absence of detailed quantitative information [96]. Hence, we also employed this approach to study cell fate decision using a MATLAB-based toolbox. The model enables us to map environmental signals to describe the relation between the intracellular signaling molecules and cell state. A detailed description of the Boolean network modeling and its application in biological systems is outside the scope of this paper, but is presented in reference [96].

### Cellular potts model—Cellular level

An agent based cellular Potts model is employed to simulate the dynamics of the cellular system. The model is a discretized lattice Monte Carlo developed by Glazier and Graner [97] that employs spatially extended generalized cells. Generalized cells not only can be representative of single cells and their compartments but can also represent clusters of cells. This flexibility permits to take into account different levels, from intracellular to tissue scale. The approach enables us to capture behaviors such as growth, proliferation, migration, and apoptosis of cancer cells and to determine ECs reaction to the tumor-induced conditions.

To create the modeling framework, the computational domain is occupied with diverse biological entities including cancer cells at different sites, ECM, and endothelial cells. In the algorithm, each cell type is identified by a unique number or index, *τ*, which is assigned to every entity occupying a lattice site (0 is assigned to all lattice sites that are filled by ECM). One unique value, *σ* = 1,2,… represents each cell (which consist of several pixels). The model is based on energy minimization, and the corresponding energy of each configuration is referred to as the Hamiltonian value, H. Therefore, the evolution of lattice sites occupation is simulated using the Metropolis criteria based on the Hamiltonian value variation: when the energy of system decreases with a pixel change, the model accepts the pixel swap, following the minimization algorithm; otherwise the update is only accepted with a Boltzmann probability, $e^{-\Delta H/T_{m}}$, where *T*_*m*_ is a parameter representing the level of cell-membrane fluctuations, describing the effective cell motility.

The Hamiltonian that controls cellular dynamics is the sum of four terms that describe cell-cell adhesion, cell growth, chemotaxis and guarantee cell continuity.

One of the key biological and physical properties of cells is the contribution of cell adhesion at the cell membrane. The adhesion energy is an important property that is required for tissue integrity and cell motility and establishes the interaction between adjacent cells as well as between cells and the ECM. It is also required for tissue integrity and cell motility. Since this energy is a cell type dependent energy, it is based on the coupling strength between the entities of types *τ* and *τ*^*’*^, *J*_*τ*,*τ’*_, as follows:

$$E_{\text{adhesion}}=\sum_{\text{site}}J_{\tau ,\tau \prime }(1-\delta_{\sigma ,\sigma \prime })$$ (1)

where the sum is run over all the neighboring pixels, *σ*, *σ’* the cells’ ID, and *δ* is the Kronecker symbol.

The second key property considers the required energy to maintain cell size and describe cell growth during a cell cycle. During the cell cycle the cells double their size before undergoing cell division. After mitosis, the daughter cells inherit the same phenotype, and one of the resulting daughter cells keeps the parent cell ID and the other daughter cell gets a new unique ID. The following equation describes the term in the Hamiltonian responsible for setting the target cell size:

$$E_{\text{growth}}=\sum_{\text{cell}}\gamma_{e}{\left(a_{\sigma }-A_{\sigma }^{T}\right)}^{2}$$ (2)

Thus, *a*_*σ*_ denotes the current area of cells and $A_{\sigma }^{T}$ is the target area (double of the quiescent area for proliferating cells) and *γ*_*e*_ is the cell elasticity.

Since cells are continuous structures, with all the pixels in contact, it is necessary to keep the continuity of lattice sites that are occupied by a single cell. To preserve the connectivity of pixels of each individual cell, a special term is considered in the Hamiltonian. This term is a constraint that once the cell is to rupture, the total energy of system is drastically increased,

$$E_{\text{continuity}}=\sum_{\text{cell}}\alpha (1-\delta_{a_{\sigma },{a\prime }_{\sigma }})$$ (3)

where *a*_*σ*_ is the current cell size and $a_{\sigma }^{\prime }$ is the number of continuous lattice sites that are occupied by the cell with unique identification *σ*. With a difference between *a*_*σ*_ and $a_{\sigma }^{\prime }$, *α* boosts the system energy by a large value.

Chemotaxis of tumor cells in their surrounding environment is a key component of cancer progression and metastasis. Recent research in the study of tumor cells chemotaxis have reported different modes of cell migration (see review [98]). Migration of tumor cells towards blood vessels is a commonly observed phenomenon; hence, assuming nutrients and oxygen diffused from vessels as a chemoattractant, tumor cells with migration phenotype move by chemotaxis toward the vessels. The following is the energy that is involved in chemotaxis of migrating cells, proportional to the nutrients concentration (*n*) gradient:

$$E_{\text{chemotaxis}}=\sum_{\text{cell}}\chi_{\sigma }\Delta n$$ (4)

Moreover, in what concerns sprouting angiogenesis, migration of endothelial cells is the most important event. VEGF is the main regulator of vascular network development, known as a chemoattractant agent for ECs. The gradients of VEGF concentration induce the ECs to move towards higher concentration (*V*) values,

$$E_{\text{chemotaxis}}=\sum_{cell}\chi_{\sigma }\Delta V$$ (5)

where *χ*_*σ*_ is a parameter standing for chemotaxis intensity.

Haptotaxis, the directed motion of cells according with the concentration gradient of a molecule linked to the ECM, is essentially modeled through the adhesion energy between adjacent cells and the surrounding microenvironment [26].

Therefore, in order to model the interactions of tumor cells in the ECM, the Hamiltonian is the contribution of the energy terms for adhesion, area, continuity and chemotaxis, as follow:

$$H=E_{\text{adhesion}}+{E_{\text{growth}}+E}_{\text{continuity}}+E_{\text{chemotaxis}}=\sum_{\text{site}}J_{\tau ,\tau \prime }(1-\delta_{\sigma ,\sigma \prime })+\sum_{\text{cell}}\gamma_{e}{\left(a_{\sigma }-A_{\sigma }^{T}\right)}^{2}+\sum_{\text{cell}}\alpha (1-\delta_{a_{\sigma },{a\prime }_{\sigma }})+\sum_{cell}\chi_{\sigma }\Delta c$$ (6)

where the chemoattractant *c* is the nutrient concentration *n* for the tumor cells and the VEGF concentration *V* for the activated endothelial cells.

### Reaction- diffusion equations—Extracellular scale

During tumor growth, cells that undergo oxygen deprivation (hypoxia), release angiogenic factors, such as VEGF, to access nutrients by stimulating the growth of new capillaries [99]. To model this process, a diffusion-reaction equation is employed to describe the diffusion of nutrients from vessels into the microenvironment, as follows,

$$\frac{\partial n}{\partial t}=D_{n}\nabla^{2}n-B(x,y,n)+S_{n}$$ (7)

where *n* is nutrient concentration, *D*_*n*_ is the diffusion coefficient of *n* and *S*_*n*_ is the term for release of nutrients from vessels. *B* is a function that refers to the uptake of nutrients by cancer cells, as described below:

$$B(x,y,n)=\{\begin{array}{c} n\;\text{if}\;0\leq n\leq \beta \;\text{and}\;\{(x,y)\subset \text{Cancer}\;\text{cell}\}\\ \beta \;\text{if}\;n>\beta \;\text{and}\;\{(x,y)\subset \text{Cancer}\;\text{cell}\}\\ 0\;\text{if}\;\{(x,y)⊄\text{Cancer}\;\text{cell}\} \end{array}$$

*β* is the maximum amount of nutrients that a cancer cell can consume. The value of β is different for each tumor cell phenotype. It should be noted that the normal healthy cells also consume nutrients, but with a different rate of uptake. Moreover, the release rate of nutrients is only different from zero for the endothelial cells, i.e.:

$$S_{n}(x,y)=\{\begin{array}{c} s_{n}\;\text{if}\;\{(x,y)\subset \text{Endothelial}\;\text{cell}\}\\ 0\;\text{if}\;\{(x,y)⊄\text{Endothelial}\;\text{cell}\} \end{array}$$

The simulation starts with four tumor cells at the center of computational domain. The concentration of nutrients inside the domain is sufficient, and their diffusion from vessels provide essential nourishment to keep the signaling from RTK receptors active. So, initial and boundary conditions are imposed as: *n*(*x*, *y*, *t*)|_(*x*,*y*)∈EC*s*_ = *S*_*n*_, *n*(*x*, *y*, 0) = *S*_0_ = 4.6 pg/voxel, with periodic boundary conditions.

The secretion of VEGF generates a concentration gradient between the tumor and the nearby vascular network and, consequently, activates the endothelial cells. VEGF distribution is described, in a similar way, by a partial differential equation (PDE). Considering diffusion, decay, and uptake of the VEGF, the final equation is:

$$\frac{\partial V}{\partial t}=D_{V}\nabla^{2}V-kV-E(x,y,n)+S_{V}$$

$$E(x,y,V)=\{\begin{array}{c} V\;\text{if}\;0\leq V\leq e\;\text{and}\;\{(x,y)\subset \text{Endothelial}\;\text{cell}\}\\ e\;\text{if}\;V>e\;\text{and}\;\{(x,y)\subset \text{Endothelial}\;\text{cell}\}\\ 0\;\text{if}\;\{(x,y)⊄\text{Endothelial}\;\text{cell}\} \end{array}$$

$$S_{V}(x,y)=\{\begin{array}{c} s_{V}\;\text{if}\;\{(x,y)\subset \text{Hypoxic}\;\text{cancer}\;\text{cell}\}\\ 0\;\text{if}\;\{(x,y)⊄\text{Hypoxic}\;\text{cancer}\;\text{cell}\} \end{array}$$ (8)

where *k* is the of decay rate of VEGF (*V*), *D*_*V*_ is the diffusion constant, *S*_*V*_ is the secretion rate of VEGF, whereas function *E* denotes the uptake of VEGF by ECs, with the maximum amount of *e*. Due to the limitation in access to the nutrients, the hypoxic cells inside the tumor up-regulate pro-angiogenic signals, including VEGF. Hence, in the initial conditions of the simulation, there is no VEGF in the domain until the hypoxic core of the tumor is formed and signaling from growth factors are activated, which means *V* (*x*, *y*, 0) = 0. The hypoxic tumor cells start to release VEGF field with a rate of *s*_*V*_, taken from experimental data, and then it is available within the tumor core.

### Boolean network model and signaling cascade incorporation

To understand the details of cells response to the signals received from their external environment, we used a Boolean model framework to describe the tumor growth process and therapeutic approaches. The model focuses on pivotal pathways involved in tumor development, by investigating the cross-talk between receptor tyrosine kinases (RTKs) and the growth factors involved, cell-cell and cell-matrix communication controlled by integrin and cadherin, and the key cascade regulating cancer development, the Wnt signaling pathway. It infers the cell phenotype, whether it is growing, migrating, or undergoes apoptosis once signals are activated, by coupling the intracellular with the cellular level of our multiscale technique.

**Fig 3:**
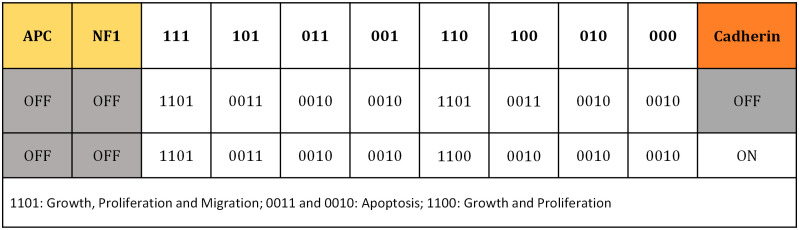
Boolean model prediction of cell phenotype (state) for various input configurations. Colors correspond to the color of the nodes in Fig 2, indicating activity of the receptors’ signal (i.e. integrin, RTK, Wnt) and inactivation of receptor is shown in grey. Tumor suppressors are deactivated. For instance, if the cell receives a signal from integrin and RTK, and no signal from Wnt (110), considering cadherin activation, the model predicts that the cell starts to grow and proliferate (1100). The binary code on the first row specify the integrin, RTK and Wnt states, respectively.

In the current Boolean network model, a *n* binary state exists for *n* variables, {*x*_1_. *x*_2_. ⋯. *x*_*n*_}, which can take the values 1 and 0 indicating “on-off” regulatory switches. Considering *x*_*i*_ as the state of a particular node, it can be regulated by *k* other input elements, with 0 ≤ *k* ≤ *n*, whereby *x*_*i*_ can regulate *j* other output elements, with 0 ≤ *j* ≤ *n*. All network elements are connected to each other by a set of Boolean regulation functions, *F* = {*f*_1_. *f*_2_. ⋯. *f*_*n*_}, represented by a look-up table. Once inputs variables have been assigned, the state of the node network elements are updated according to their corresponding Boolean functions: *x*_*i*_(*t* + 1) = *f*_*i*_(*x*_*i*1_(*t*), *x*_*i*2_(*t*), …, *x*_*ik*_(*t*)). Finally, an input/output table is derived, which predicts role of the external cues in cell phenotype determination (Fig 3).

Incorporating intracellular regulation to the model is an important step toward modeling a biological system in a realistic way, and provides the possibility to investigate how the external environment controls the cells behavior, and how it directs tumor progression. Thereby, our multi-scale model integrates the extracellular level in which partial differential equations describe VEGF and nutrients dynamics, while the cell-based stochastic model represents the cell dynamics. This depends on the Boolean network model that depicts the involvement of the signaling transduction pathways.

Eliminating the common prescribed rules, the cellular phenotype predicted by the intracellular analysis is introduced into the model and the cellular behavior is based on the microenvironment conditions. Hence, each cell gathers information on the nutrients and VEGF concentration, and explores the connection with the adjacent cells and matrix fibers and molecules, at each time step. Then, these environmental cues are applied via the signal transduction network and interpreted by the cell, and finally determine the cell phenotype, according to the table in Fig 3.

To measure the signal strength from RTK receptors, the available experimental data is used, based on the quantity of VEGF that an EC can bind to get activated, and the critical nutrients concentration limit for surviving hypoxic conditions. However, there is a lack of experimental data on the strength of integrin, cadherin and Wnt receptors, since their activity is strongly dependent on the dynamics of cell’s environment. To apply the signaling from these receptors, we estimate their strength by assessing the cell-ECM and cell-cell contact, normalized by the cell’s size. Any differences in signal strength are interpreted by the cells and influence their state. After the determination of each cell phenotype, cells phenotype is assigned and introduced in the cellular model. Parameter calibration is done through a sensitivity analysis. Then the system behavior evolves through Monte Carlo steps and toward energy minimization. The new cells’ states, and nutrients and VEGF concentrations are inputs for the next iteration (time step).

### Simulation algorithm

The model couples multiple time scales by connecting each level and considering the feedback of a specific level on the next one. After receiving signals from the extracellular environment, the cell’s phenotype is determined at the intracellular level by the activated signaling pathways. The possible tumor cell phenotypes are growth, proliferation, migration, quiescence and apoptosis. The simulation of tumor evolution then moves to the cellular level whereby the cell state is used to track the changes from the system energy variation (through Eq 6). The predicted cell phenotype at the intracellular scale is implemented via the Eq 6, in which parameters are different for each phenotype. When the signaling is processed and applied at the cellular level, the new topology of tumor, and distribution of nutrients and VEGF are determined and implemented as inputs to the next iteration. This is repeated and the constantly changing environment controls the biological responses of cells and leads to the tumor development simulated with the Metropolis criteria.

It should be noticed that the process is repeated for each pixel, randomly chosen, in the cell lattice at each Monte-Carlo step (MCS). Since MCS and experimental biological scale of time are related, in the current model it is assumed that 1 MCS represents one real time minute based on the fastest cell cycle time for cell division ~24h [26,38].

### Implementation parameters

Our model is developed through the open-source CompuCell3D simulation environment (http://www.compucell3d.org/). The size of the modeled lattice is 300 × 300 × 1, equivalent to 1.44mm^2^, and the average size of a tumor cell is approximately 500μm^2^. Periodic boundary conditions are considered for the lattice domain.

The simulation starts with 4 proliferating tumor cells and different tissue structures around them. We assume that nutrients, particularly oxygen, are diffused from the pre-existing and tumor induced vascularization, and its diffusion constant is approximately half of the diffusion constant of oxygen in the water.

It is assumed that if the available nutrient concentration within the cancer cells is below a specified threshold, it activates the signaling PI3K-Akt pathway recruited by RTK. According to the results of our Boolean model, inactivation of RTK receptor signaling, leads to cell apoptosis. Hence, cells undergo apoptosis below the corresponding threshold that is defined from the rate of nutrients consumption normalized by the cell target area. To consider the activation of signaling from integrin, E-cadherin and Wnt, the thresholds for the turn-on of each receptor, are defined from the connection of cells with their neighbor cells and ECM to track the effective motility, contact inhibited growth and, finally, tumor morphological changes. Similarly, a threshold is defined for the concentration of VEGF to activate the ECs and make them respond to the angiogenic factors secreted by the hypoxic tumor cells. Above the threshold level, ECs become activated and angiogenesis begins.

We assume that the normal healthy cells are on a homeostatic situation in the absence of tumor and have adequate nutrients availability. Due to high oxygen consumption by viable (non-necrotic) tumor cells in comparison with the healthy surrounding tissue cells [100], the rate of oxygen uptake by tumor cells is considered 3 times the consumption rate for normal cells. Viable tumor cells can be in one of three different states: quiescent, proliferating and migrating. To set the rate of oxygen consumption per unit cell for these cell states, we used the experimental data reported by Freyer [101]. All parameters and properties of the fields are given in Table 2.

**Table 2:** Parameters used in the model and corresponding references.

Parameter	Symbol	Value	Ref.
**Nutrients Diffusion Equation Parameters**			
Nutrient diffusion constant	*D*_*n*_	10^3^ μm^2^/s	[12]
Nutrient Source	*S*_*n*_	8.83×10^−16^ mol/cell/s *	[8]
Nutrient consumption of proliferating and migrating cells	*β*_*P*_	5.17×10^−17^ mol/cell/s *	[101]
Nutrient consumption of quiescent cells	*β*_*Q*_	2.41×10^−17^ mol/cell/s *	[101]
Nutrient consumption of necrotic cells	*β*_*N*_	0.00 mol/cell/s	
RTK Signal Threshold	*T*_*RTK*_	4.48×10^−3^ pg/pixel	[102]
Integrin Signal Threshold	*T*_*ITG*_	0.3	Estimated
Cadherin Threshold	*T*_cadherin_	0.3	Estimated
Wnt Threshold	*T*_*Wnt*_	0.15	Estimated
**VEGF Diffusion Equation Parameters**			
VEGF Diffusion constant	*D*_*V*_	10 μm^2^/s	[103]
VEGF decay	*k*	0.9375 h^−1^	[103]
VEGF uptake	*e*	0.001 pg/cell/s *	[104]
VEGF Source	*s*_*V*_	0.035 pg/pixel	[105]
Activation threshold	*T*_*V*_	0.00095 pg/pixel	[26]
**Cellular Potts Model Parameters**			
Migrating cells elasticity	*γ*_eM_	8	Estimated
Proliferating cells elasticity	*γ*_eP_	8	[106]
Quiescent cells elasticity	*γ*_eQ_	8	Estimated
EC membrane elasticity	*γ*_eEC_	8	Estimated
Intracellular Continuity	*α*	300	[26]
Boltzmann Temperature	*T*_*m*_	10	Estimated
Cell-Cell adhesion Matrix	$J=[\begin{array}{l} J_{\text{EC-EC}}\;J_{\text{M-EC}}\;J_{\text{P-EC}}\;J_{\text{Q-EC}}\;J_{\text{N-EC}}\;J_{\text{m-EC}}\\ J_{\text{EC-M}}\;J_{\text{M-M}}\;J_{\text{P-M}}\;J_{\text{Q-M}}\;J_{\text{N-M}}\;J_{\text{m-M}}\\ J_{\text{EC-P}}\;J_{\text{M-P}}\;J_{\text{P-P}}\;J_{\text{Q-P}}\;J_{\text{N-P}}\;J_{\text{m-P}}\\ J_{\text{EC-Q}}\;J_{\text{M-Q}}\;J_{\text{P-Q}}\;J_{\text{Q-Q}}\;J_{\text{N-Q}}\;J_{\text{m-Q}}\\ J_{\text{EC-N}}\;J_{\text{M-N}}\;J_{\text{P-N}}\;J_{\text{Q-N}}\;J_{\text{N-N}}\;J_{\text{m-N}}\\ J_{\text{EC-m}}\;J_{\text{M-m}}\;J_{\text{P-m}}\;J_{\text{Q-m}}\;J_{\text{N-m}}\;J_{\text{m-m}} \end{array}]=[\begin{array}{cccccc} 5 & 30 & 30 & 30 & 30 & 12\\ 30 & 8 & 8 & 8 & 10 & 12\\ 30 & 8 & 8 & 8 & 10 & 12\\ 30 & 8 & 8 & 8 & 10 & 12\\ 30 & 10 & 10 & 10 & 8 & 10\\ 12 & 12 & 12 & 12 & 10 & 66 \end{array}]$		

*Each tumor cell has an initial volume of about 32 voxels, with an equivalent value used in our calculations.

### Results and discussion

#### Cell phenotype change

The determination of cell phenotype in response to the various external stimuli is classified in an input-output table. This map determines the cellular phenotype through the possible input combinations in the signaling network (Fig 3). In this table the states 1 and 0 correspond to on and off switches for activation of each component.

The arrangement of the inputs is divided in three parts, in which the activity of the receptors integrin, RTK and Wnt, is set across the top of the table and the signal from cadherin is monitored exclusively, because of its key role in controlling cell-cell communication and association to cancer invasion. Normally, tumor-suppressors have a key role in cell cycle progression and are involved in cells’ apoptosis. However, loss of function of these proteins would result in increased cell proliferation and consequently to cancer. As the purpose of this study is to explore tumor evolution, it is assumed that there is a deficiency of tumor suppressors, APC and NF1, which means they have no role in promoting apoptosis. Finally, each condition leads to a distinct cellular response, these being “cell growth, proliferation and migration”, “cell apoptosis” and “cell growth and proliferation” (Fig 3). For instance, if the cell receives a signal from integrin and RTK, and no signal from Wnt (110), considering cadherin activation, the model predicts that the cell starts to grow and proliferate (1100).

Consistent with several experimental observations, disruption in the activity of either RTK or integrin receptors lead to cell apoptosis, independent from cadherin activity [107–110]. Interestingly, cadherin signaling regulates contact inhibition, that controls cell motility, revealing its role in cell–cell communication. Moreover, results show that in absence of Wnt signaling, cadherin may block cell migration (case 110). Importantly, tumor cells are able to reach the quiescent state when they become oxygen starved and hypoxic. Cells in a quiescent state stop growing and proliferating.

Incorporating these results in our multiscale model of tumor development, it takes into account the microenvironment regulation of cell function and thus the model represents a system that describes the biology-based tumor behavior. Including important signaling pathways also helps to explore therapeutic strategies by capturing inhibitor targets that control cell proliferation and induce cell apoptosis. We will investigate how disruption in receptors activity leads to tumor shrinking and promotes the suppression of tumor growth. More details on the model validation are presented in the following section.

### Validation of Boolean model of intracellular signaling pathways—The robustness of signal transduction

To ensure whether the information of the proposed input/output map is predicting unique points and external inputs singly determine the foresee phenotypes, we simulated all possible 2^29^ initial combinations of internal node states. In all cases, the network dynamics converge to the same attractors shown in Fig 3, and prove the strong robustness of the corresponding signal transduction against fluctuations. Back to the phenotype map, the presence of both integrin and RTK signals is essential for cell survival, specifying the pivotal role played by crosstalk between these receptors. Our results indicate that if either receptor activity is disrupted, apoptosis is induced, and several experimental observations confirm this result [107–112]. Moreover, the simulated network exhibits cell apoptosis when the signaling pathways Ras/Raf/MEK/ERK and PI3K/PTEN/Akt/mTOR, are inhibited, which is confirmed by [69,113]. We used this result in targeted therapy.

To validate our multiscale model, experimental data from an *in vivo* model is used as a recognized benchmark, in which vascular events after corneal implantation of a rabbit tumor were followed [114,115]. According to the reported results, the initial growth pattern is linear and sprouts extend at a rate of approximately ∼0.50 mm/day, with a measurement uncertainty of 0.1mm/day, which corresponds to an extension velocity of 21 ± 4 μm/h. Then the sprout progression proceeded at reduced average speeds, between 0.25−0.50 mm/day [114]. A quantitative comparison of extension speeds between our simulation results and experimental data shows a good agreement (Table 3). Estimated vascular extension speeds are an average of 5 independent simulations, with the same parameter set, recording the total sprout displacement with time. We also compared our results with the more recent experimental measurements by Kearney et al. [116]. They analyzed the dynamics of vascular sprout formation from embryonic stem cells and reported an average extension speed of new sprouts of 14 μm/h, in a period of 10 hours. The corresponding average velocity in our model is estimated at ∼13 μm/h.

**Table 3:** Quantitative comparison of simulated average sprout velocity with various experimental observations.

**Average Sprout Velocity (μm/h)**	**Experimental Reports [114]**		**Simulation Results (In Average)**	
	**Initial**	**In progress**	**Initial**	**In progress**
	21 ± 4	10± 4	25±3.7	11±1.2
	**Experimental Reports [116] (Averaged in 10h)**		**Simulation Results (Averaged in 10h)**	
	14		13±1.6	

**Fig 4:**
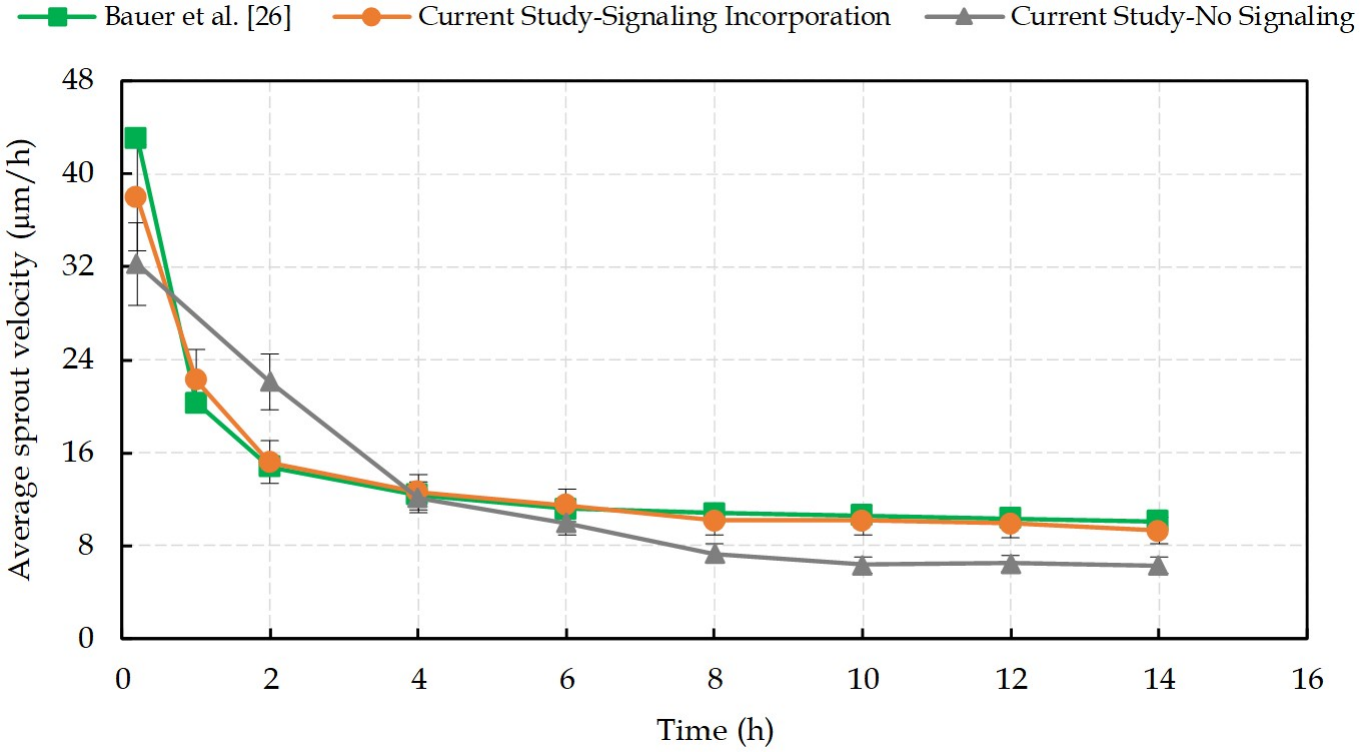
The average sprout extension velocity calculated from 5 independent simulations with and without including the intracellular signal transduction pathways, in comparison with experimental measurements extracted from Bauer et al. [26]. (Model validation; the error bars are the standard deviation on the mean of n = 5 simulation runs).

Fig 4 shows the simulated average sprout extension velocity as a function of time, in comparison with the results presented by Bauer et al. [26], extracted from the experimental reports. According to them, linear vessels growth within the first two hours proceeded at a higher average velocity (∼25 μm/h) and as the sprout develops and new proliferating ECs form the vessel structure, the cellular adhesion to the ECM and the cell-cell adhesion decrease the extension speed.

Numerical results are obtained for two cases, namely with and without introducing the intracellular signal transduction pathways. The role of signaling pathways in determining the cell-cell and cell-ECM adhesion is clearly evident when the intracellular signaling in sprout development is not considered. The accurate prediction by our model relies on the intracellular scale, which is based on the Boolean network approach. The comparison between the results of the model with and without the intracellular processes not only shows the importance of the signal transduction pathways contribution to modeling cellular regulation, but also justifies that the Boolean network model is able to capture the essential features of biological processes in angiogenesis and tumor growth.

The classic Michaelis-Menten model for the cells’ oxygen consumption rate seems to be a more realistic description of nutrients use [117–119]. So, in order to check if there is any inconsistency with the current approach, we compared our results with the numerical simulations of tumor growth presented by Taghibakhshi et. al [119], in which the oxygen consumption is approximated by Michaelis-Menten kinetics, as follow:

$$R_{\text{P}}=\frac{V_{max}n}{K+n}$$ (9)

$$R_{\text{Q}}=\frac{1}{2}\frac{V_{max}n}{K+n}$$ (10)

Where *n* represents nutrients concentration, *V*_*max*_ is the maximum consumption rate of nutrients and *K* corresponds to the Michaelis constant. The authors developed the model by using a value of 0.8mM for the oxygen concentration in the culture medium. As a comparison with the present study, the oxygen consumption rates corresponding to the Michaelis-Menten parameters are summarized in Table 4.

**Table 4:** Parameters used in the models and corresponding references.

Parameters	Values	Descriptions
***V*_*max*_**	2.74 × 10^−2^ mol/m^3^/s	Maximum reaction rate [119]
***K***	4.64 × 10^−3^ mol/m^3^	Michaelis constant [119]
***R*_P_**	0.0272 mol/m^3^/s	The Michaelis–Menten nutrient consumption of proliferating cells [119]
***R*_Q_**	0.0136 mol/m^3^/s	Michaelis–Menten nutrient consumption of quiescent cells [119]
***β*_P_**	0.0252 mol/m^3^/s	Nutrient consumption of proliferating and migrating cells in Current study [101]
***β*_Q_**	0.0126 mol/m^3^/s	Nutrient consumption of quiescent cells in Current study [101]

**Fig 5:**
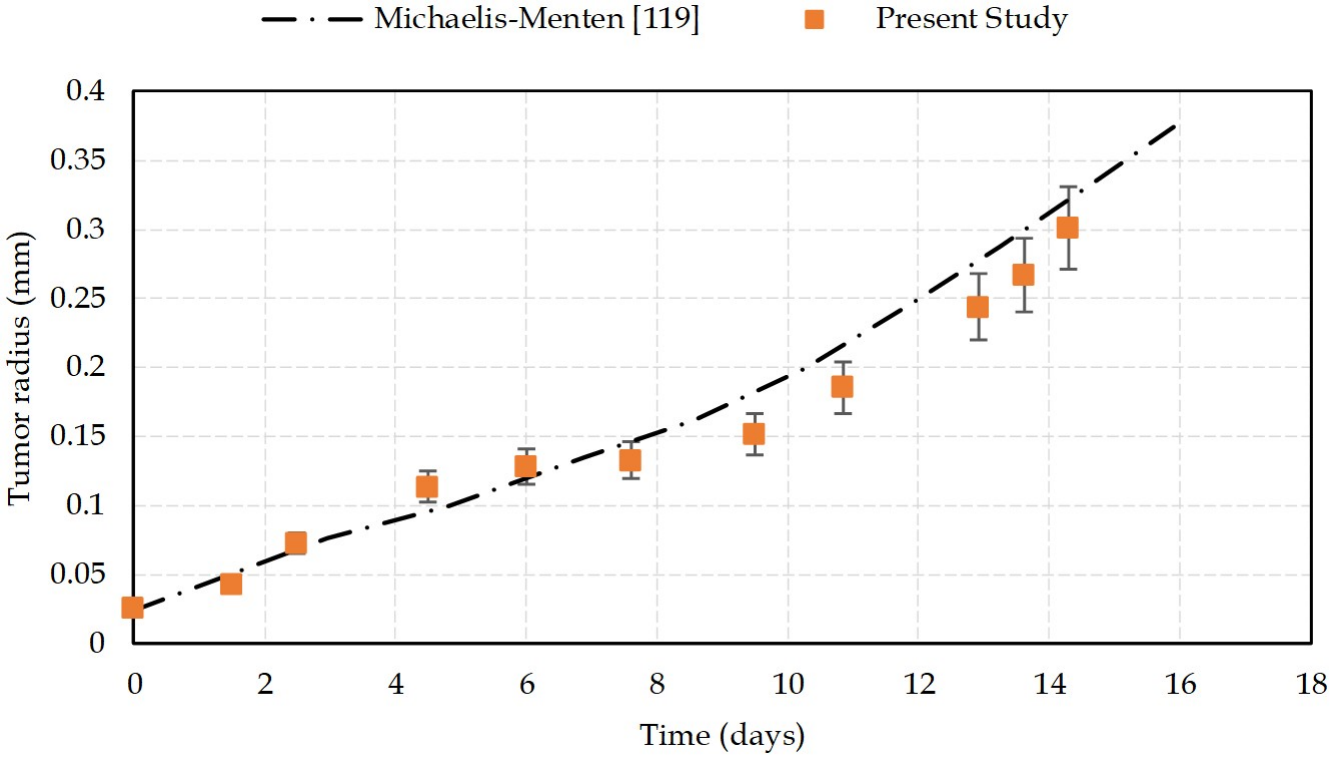
Tumor radius changes over time. A comparison between numerical results from the Taghibakhshi et al. [119] model, based on Michaelis-Menten reaction of oxygen consumption, and the current study. Initial radius of the tumor is 24.3 μm. Error bars represent standard deviations of the mean of 5 simulations.

Nutrient consumption of cells in the current study is in good agreement with the consumption rates of the Michaelis-Menten model. Here, we used the experimental data reported by Freyer [101] to model the consumption of cells according to their state. To confirm that the current study is compatible with the Michaelis-Menten reaction model, we compared our results on the evolution of the tumor radius with time, with those of the Taghibakhshi et. al [119] model (Fig 5).

Results show that there is a good agreement between both models of tumor growth simulation. Deviations originate from the different hypotheses that each model considered and implemented. The purpose of our research is to simulate the process of tumor development in a biologically realistic manner, taking into consideration a variety of events, including hyper and hypoactivation of signaling pathways and competition of cancer cells with the host environment. Nevertheless, the general behavior of tumor during its growth is fully compatible with the model in [119], which indicates that the simplification in the nutrients consumption term has no influential consequences.

### Tumor vascular growth

Simulations were run with two distinct conditions to study the process of tumor progression: tumor evolution with and without normal healthy cells.

**Fig 6:**
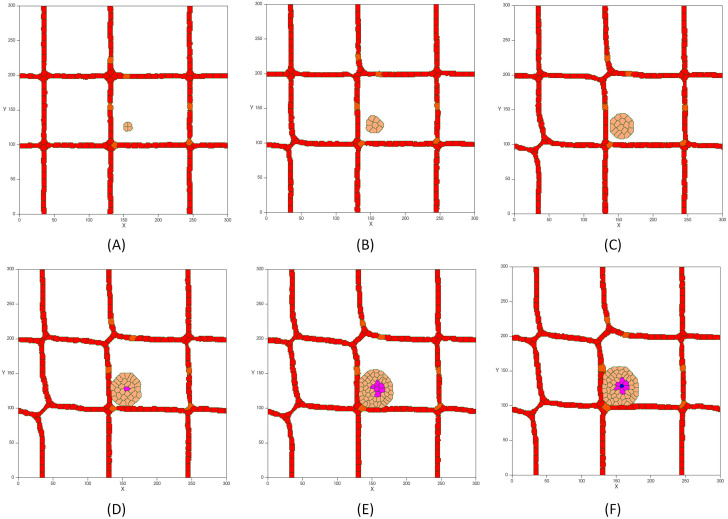
First steps of tumor avascular development in the presence of a vascular network: (A) Initial tumor with proliferating and migrating cells and a diameter of 51 μm on day 1. (B) The growing tumor reaches a diameter of 100 μm on day 2. (C) The tumor continues to grow up to a diameter of about 142μm on day 3. (D) Cells in the core of the tumor with a diameter of about 200 μm suffer from hypoxia and change their phenotype to quiescent (hypoxic cells are in purple). (E) As the tumor grows, lack of oxygen increases leading to expansion of the hypoxic core. (F) Shortage of oxygen and nutrients in the hypoxic region leads to necrosis after 20 hours on day 5 (necrotic cells are in blue).

Fig 6 shows the first steps of tumor development in presence of a vascular network without taking into account the presence of normal tissue cells. Initially, the tumor consists of four cancerous cells that have a proliferating behavior and that are able to migrate. The tumor is constantly growing for the first 5 days, while the signaling pathways, which lead to growth, proliferation and migration of cells, are activated (Fig 6A–6C). At this stage, the avascular tumor obtains nutrients by diffusion from existing blood vessels.

**Fig 7:**
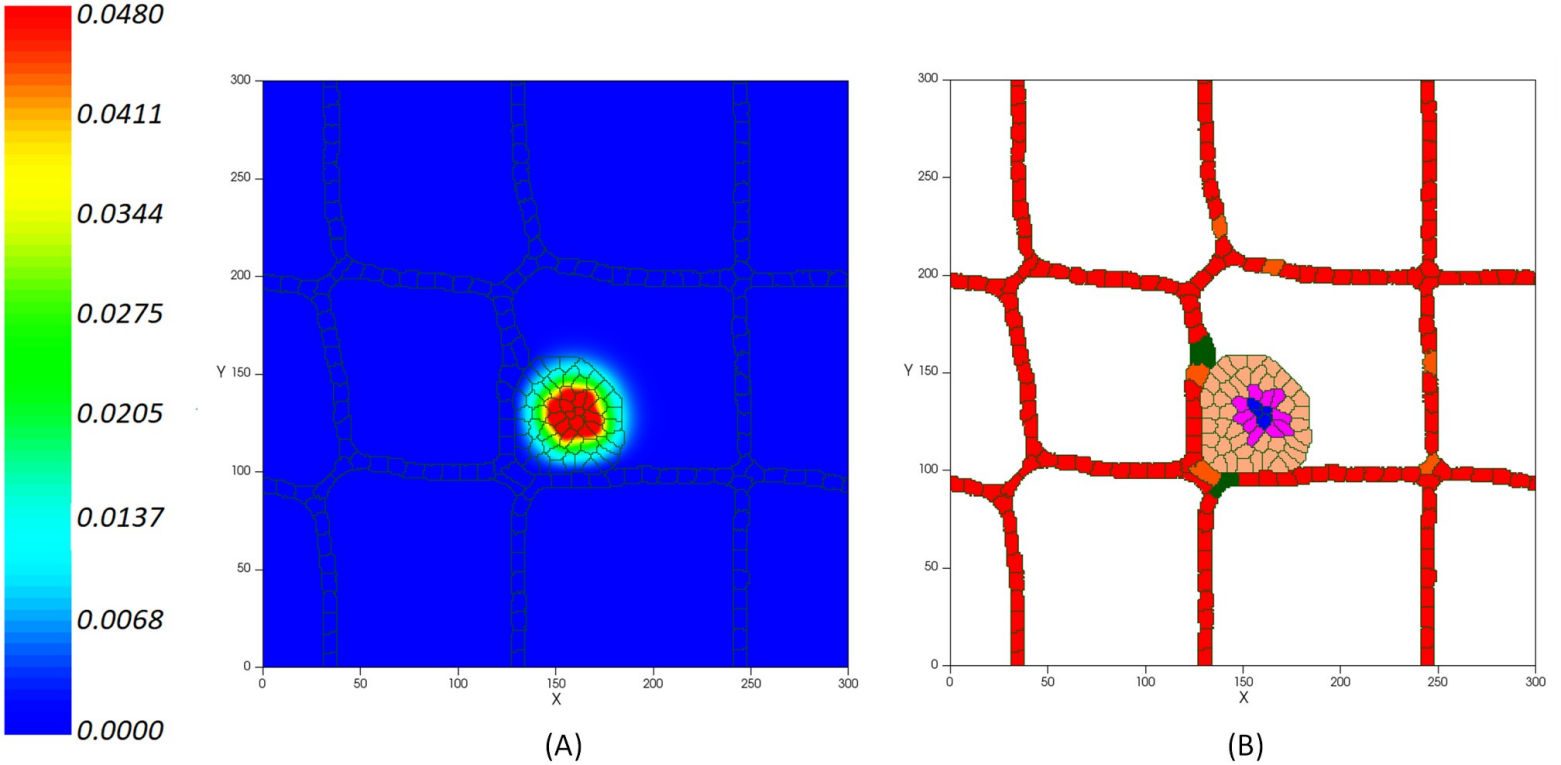
(A) Concentration of VEGF production rate per unit time (in pg/cell/s) from the tumor hypoxic core inducing the ECs’ activation. (B) Activated ECs (in green) move through chemotaxis up the gradients of VEGF.

As the tumor grows and increases in size, the need for nutrients increases. As a result, cells in the core of the tumor undergo nutrient deprivation. Simultaneously, growth factor signals are not active and cells lose the ability to uptake nutrients from the extracellular environment which ultimately leads to programmed cell death [120]. Furthermore, as the oxygen in the tumor center falls below the level needed for viability, cells become hypoxic (Fig 6D–6F). Hypoxic cells are in a quiescence state in which the cells stop growing (even though they can re-enter cell proliferation under more favorable circumstances). Once the tumor reaches a diameter of about 200 μm, quiescent cells form a hypoxic core (Fig 6D). As the tumor continues to grow, oxygen and glucose depletion affects more cells, resulting in the quiescent cells becoming necrotic (Fig 6F). At this stage, which is known as avascular growth, tumors are likely to be benign and do not invade the tissue or spread through the vessels. Since tumors need to access a supply of nutrients that ensure their growth and progress, nutrient deprived cells at the tumor core start secreting VEGF to stimulate the formation of new blood vessels (Fig 7). When VEGF reaches a nearby existing vessel, activates the endothelial cells via VEGFR1 and VEGFR2 receptors. Activated cells have the capacity to proliferate and move toward the tumor by chemotaxis (Fig 7B).

**Fig 8:**
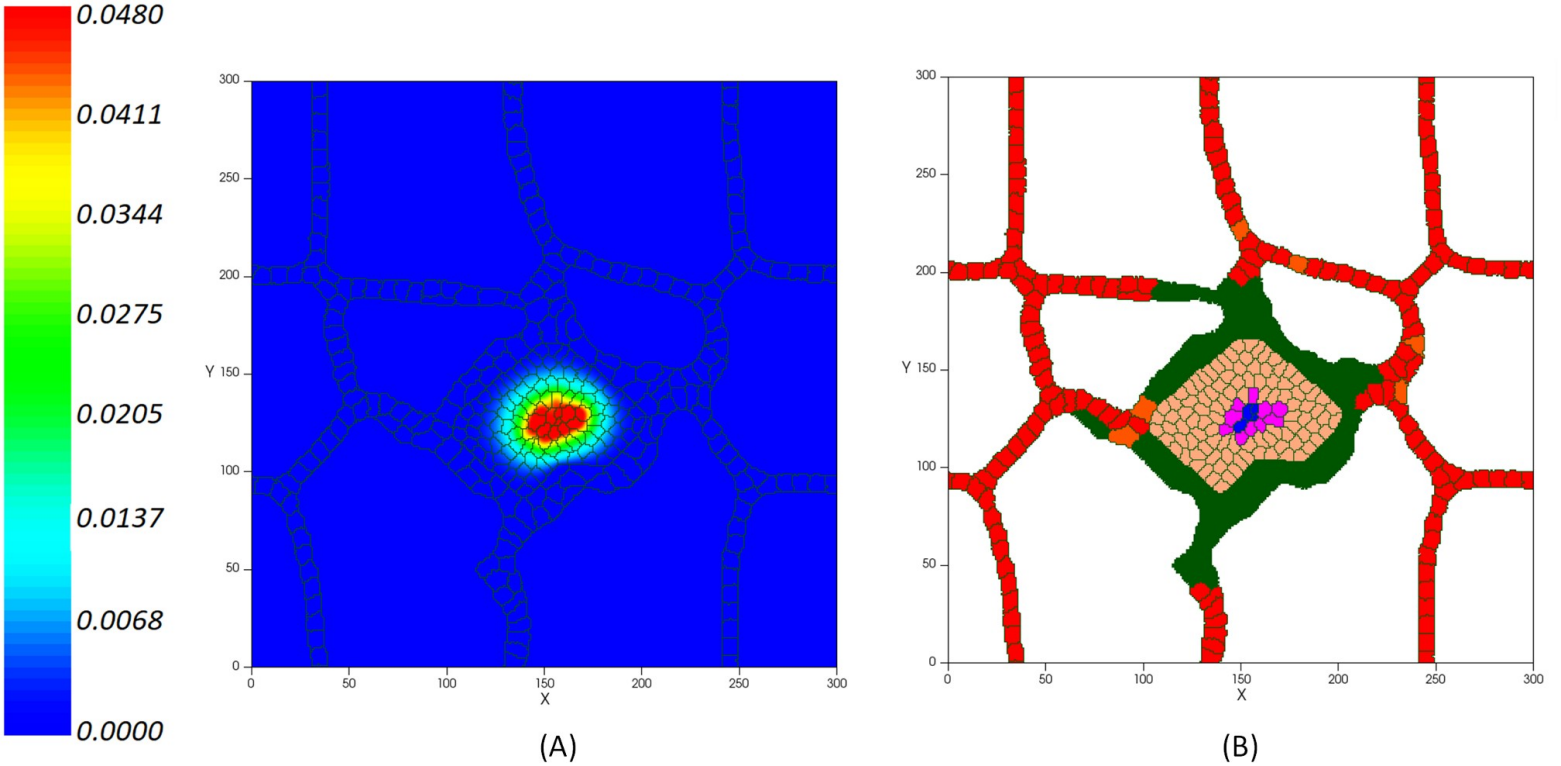
(A) Concentration of VEGF (in pg/cell/s) released from the tumor hypoxic core inducing the activation of ECs on day 12. (B) Activated ECs (in green) move through chemotaxis up the gradients of VEGF.

VE-cadherin is the main adherent junction molecule that regulates vessel formation by controlling the stability of cell membrane. It induces contact inhibition of cell’s growth by inhibiting cell’s chemotaxis response to the VEGF and blocking the proliferative response of cells [54]. Accordingly, we model the contact inhibited growth of activated cells in which, as the cell-cell adhesion junctions are increased, the cells’ growth is blocked. Therefore, it manages the coherent formation of new capillaries and prevents the growth of vessel diameter but not its length. VEGF mediates the activation of ECs when it reaches the existing vessels. As a result, ECs migrate into the extracellular matrix to reach the tumor and the new capillaries grow around the lesion. Hence, new activated endothelial cells (in green) form an orderly peripheral blood vessel around the tumor on day 12. Fig 8 shows the tumor induced vasculature. During this process, the tumor accesses the required oxygen and nutrients supply from adjacency to the new blood vessels.

**Fig 9:**
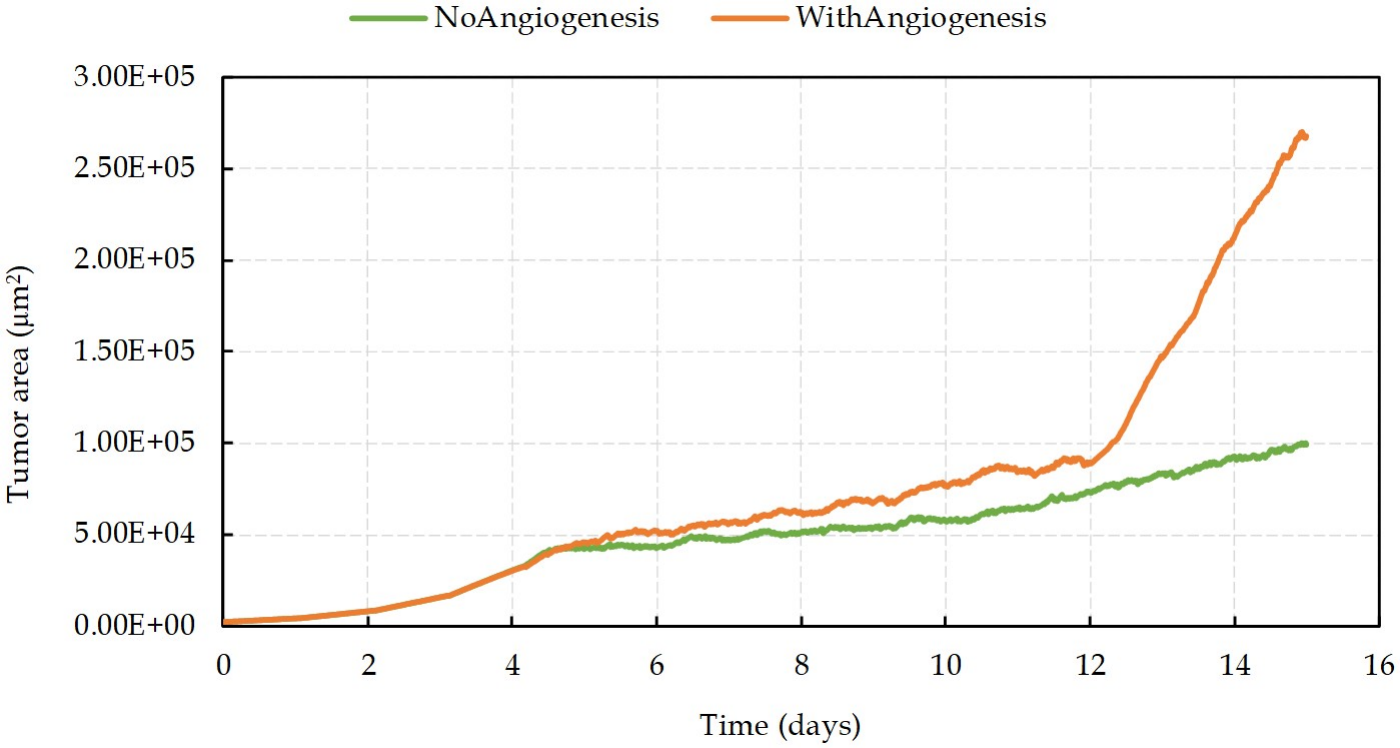
Tumor area growth rate with and without angiogenesis.

In the first 4 days and before the emergence of the nutrient-deprived condition, results show an exponential growth of tumor (Fig 9). As the hypoxic domain expands, the tumor growth slows down, while simultaneously the new vessels extend and surround the tumor (on days 4–12). As depicted in Fig 9, in contrast with the avascular tumor growth, once the sprout forms a closed loop (anastomosis, at around day 12), the rate of increase of proliferating cancer cells becomes significantly higher than without angiogenesis.

**Fig 10:**
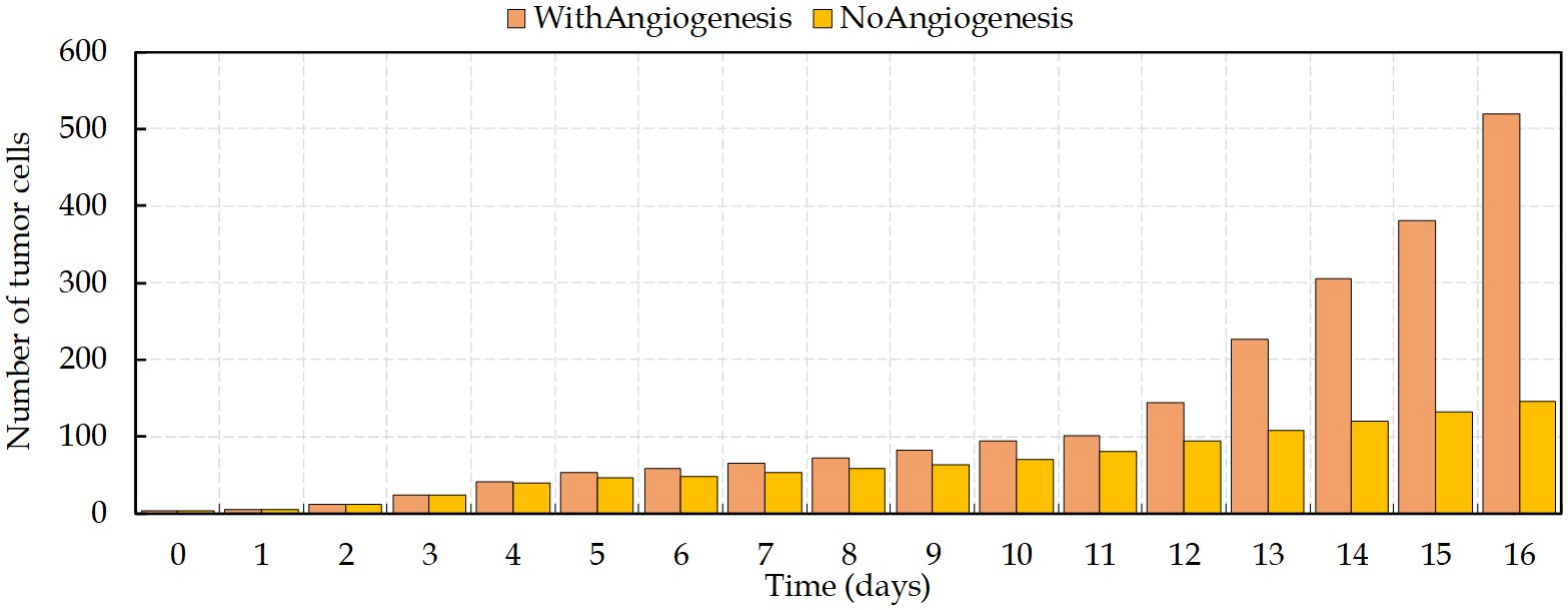
Number of viable tumor cells (non-necrotic) with time. There is an increase in the growth rate of proliferating cancer cells on day 12, when vessels surround the tumor.

Fig 10 shows the comparison between the number of viable cancer cells during the process of vascular and avascular growth. The expansion of cancer cells, starting from four cells, increases gradually before the angiogenesis. Vessels surrounding the tumor on day 12 lead to dramatic changes in the number of cells and make the tumor evolution more complex. The new disorganized vascular network supports the survival of tumor cells and improves the delivery of oxygen and nutrients. Having access to the parent vasculature can facilitate tumor cells metastasis and, since these new vessels commonly have high permeability, it increases the tumor metastatic potential [23].

### Tumor growth in presence of normal healthy cells

In contrast to normal cells and benign tumors, malignant tumors have poorly differentiated cells that can spread and invade the surrounding normal tissue. In order to supply the energy required for promotion of proliferation, survival and maintenance, tumors tend to use enormous amounts of glucose, even in the presence of oxygen. In fact, tumor cells use aerobic glycolysis, leading to what is known as the Warburg effect with the consequence of a decrease of glucose levels in the tissue and an increase in local acidity [121]. In hypoxic conditions, the activated HIF1*α* protein in cancer cells increases the rate of glycolysis and promotes the Warburg effect [122]. While hypoxia typically leads to cell death in normal cells, there is a considerably lower oxygen concentration threshold in tumor cells for which they remain viable [123]. Finally, tumors try to overcome the restrictions of nutrients availability by releasing growth factors, such as VEGF, to develop new vascularization. Furthermore, low expression of cell adhesion molecules such as E-cadherin, influences the tumor cell-cell and cell-matrix interactions. However, normal cells are firmly attached to the ECM and/or adjacent cells. Tumor cells are less adhesive in comparison with normal cells, and that makes them comparatively unrestrained. This is why malignant cells are capable of invading normal tissue to access nutrients. Considering the mentioned properties, the simulation of the tumor behavior in vicinity of normal healthy cells is presented in the following section.

### Tumor avascular growth

**Fig 11:**
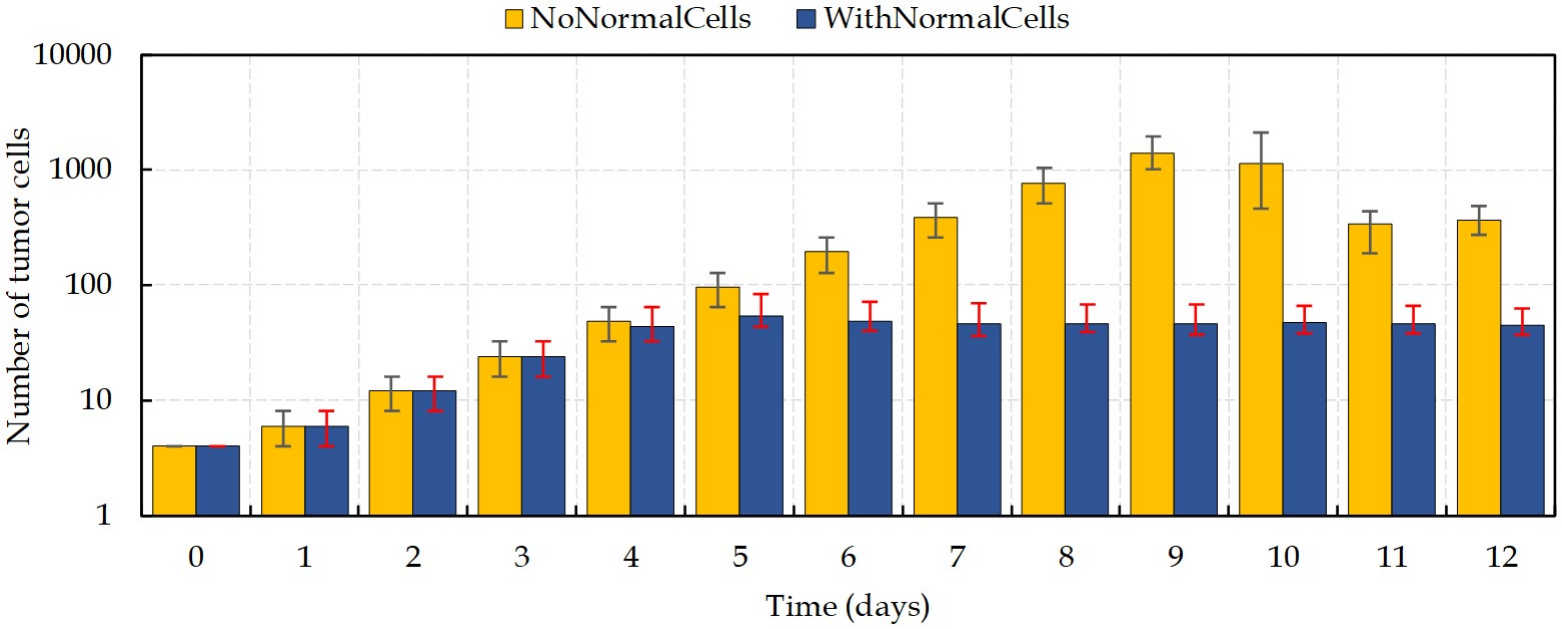
Comparison of the number of cancer cells in two different scenarios of tumor avascular growth. Bars indicate difference between maximum and minimum number of viable cells during a day. Results are the mean values extracted from four independent runs.

Fig 11 compares tumor growth with and without normal cells before the development of new vascularization. It is observed the competition between normal cells and cancer cells to access nutrients and to have enough space to grow. In the simulation with normal cells is observed the formation of a hypoxic tumor core over a relatively short period of time, (~day 4). In contrast, in the condition with no surrounding normal healthy cells, the tumor keeps growing fast until day 9. Importantly, due to the limitation of nutrients’ diffusion in the tissue, the tumor size cannot increase indefinitely.

### Tumor vascular growth

**Fig 12:**
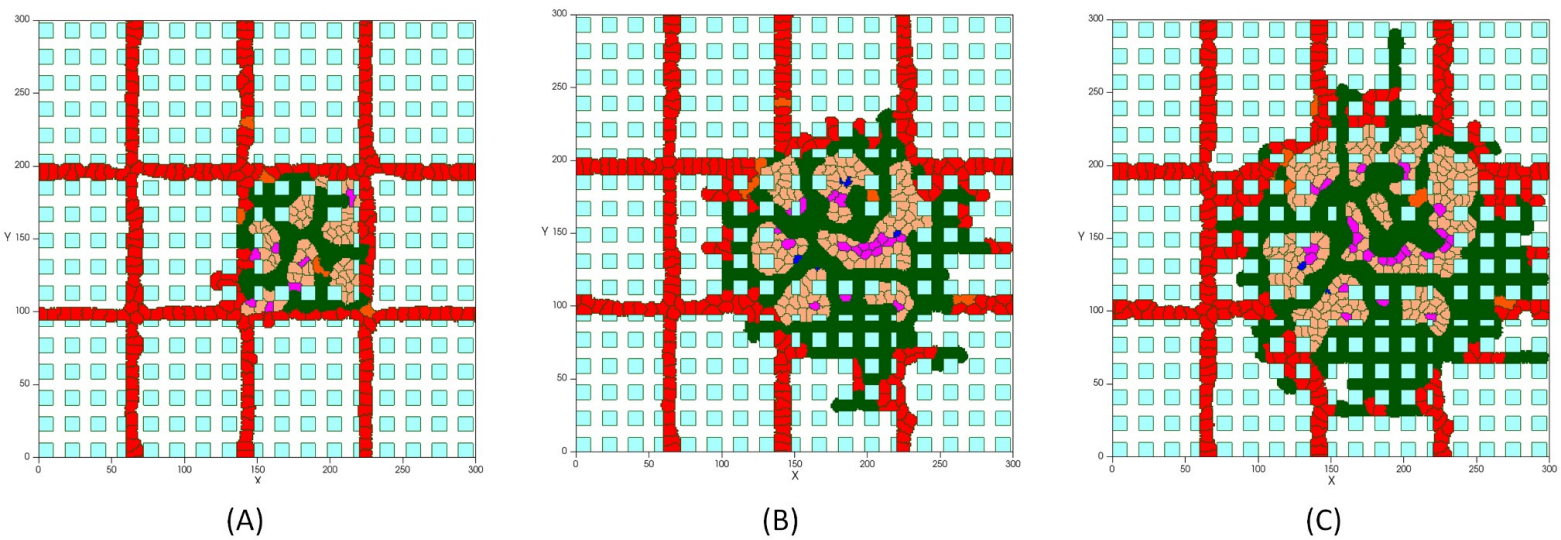
Tumor vascular growth in presence of normal healthy tissue: (A) Tumor on day 5, (B) Tumor on day 7, (C) Tumor on day 8.

In the tumor evolution with angiogenesis, the results are affected by the following two factors:

1. **Space**. Tumor cells compete both with each other and with the host normal cells for nutrients, but it is not the only relevant process that occurs. Tumor cells need to have enough room to proliferate and this is the cause of space competition between tumor and normal host tissue (Fig 12). When there is no space, the proliferation of cells in the tumor innermost areas is greatly inhibited [124]. As the growing malignant tumor invades the surrounding normal tissue, searching for space at its interface with the host cells, the host tissue mechanical resistance restrains the tumor growth dynamics. According to the simulations shown in Fig 12, as normal cells die, tumor cells expand into the vacant space. Cells in blue color are normal cells, which, over time, are disappearing from the tumor domain. Red cells represent endothelial cells that set up the preexisting vasculature, which can be distinguished in two types of inactive ECs. Both types behave in an identical way until the signaling from the RTKs activate them. The activated ECs can proliferate and move toward the tumor by chemotaxis. After mitosis of active ECs, the offspring cells are active ECs that inherit the type and target volume of the parent cell. These are shown in green. New active ECs can proliferate while the growth factors concentration exceeds the activation threshold, and the signaling transduction, via integrin and VE-cadherin receptors, regulates the migration and proliferation of cells.
2. **Intratumoral Vascularization**. Interestingly, comparing the results of tumor growth in proximity of normal cells with the case in which normal host tissue is not taken into account, a dense intratumoral vascular network is formed throughout the entire lesion, including tumor mass and surrounding normal cells, as depicted in Fig 12. The model predicts a malignant tumor growth, that contains migrating cells that can proliferate, interspersed with a high degree of neovascularization.

**Fig 13:**
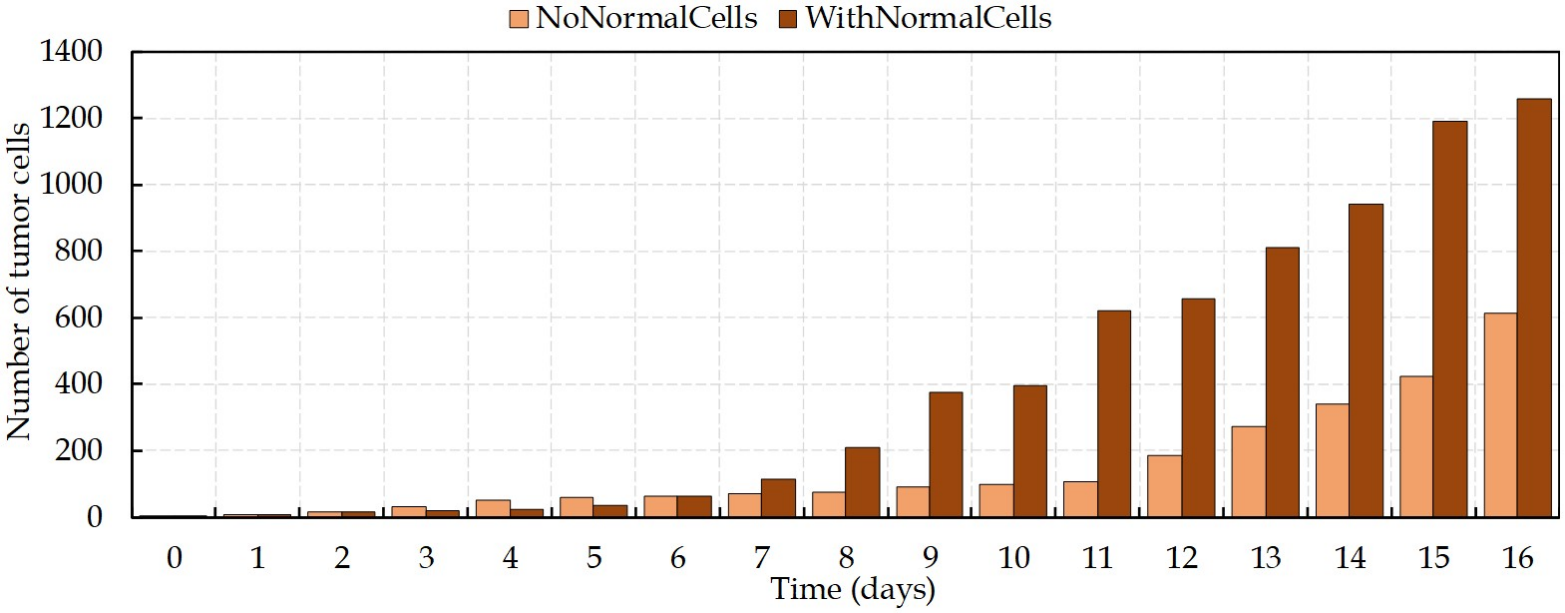
Number of tumor cells in two different conditions, with and without normal cells, of tumor vascular growth.

Accordingly, normal tissue cells at the tumor vicinity are continually exposed to hypoxic conditions, but are less tolerant to hypoxia, which ultimately lead to cell’s death. This not only leaves more oxygen to the tumor cells, but also frees up space that is readily occupied by new cancer cells. However, tumor high VEGF production promotes the growth of a dense vasculature, which supplies nutrients and increases the survival of both growing tumor cells and normal cells. It leads to a decreasing of cells’ death rate. Intriguingly, these results show that, contrary to the avascular phase of tumor growth, in the condition considering the presence of normal tissue, the daily increase in the number of cells is considerably higher than in the absence of normal cells (Fig 13). Hence, the tumor is able to reach the size of ~300μm in diameter (~620 cells) on day 11, which means the rate of proliferation is higher than when no normal tissue is considered.

**Fig 14:**
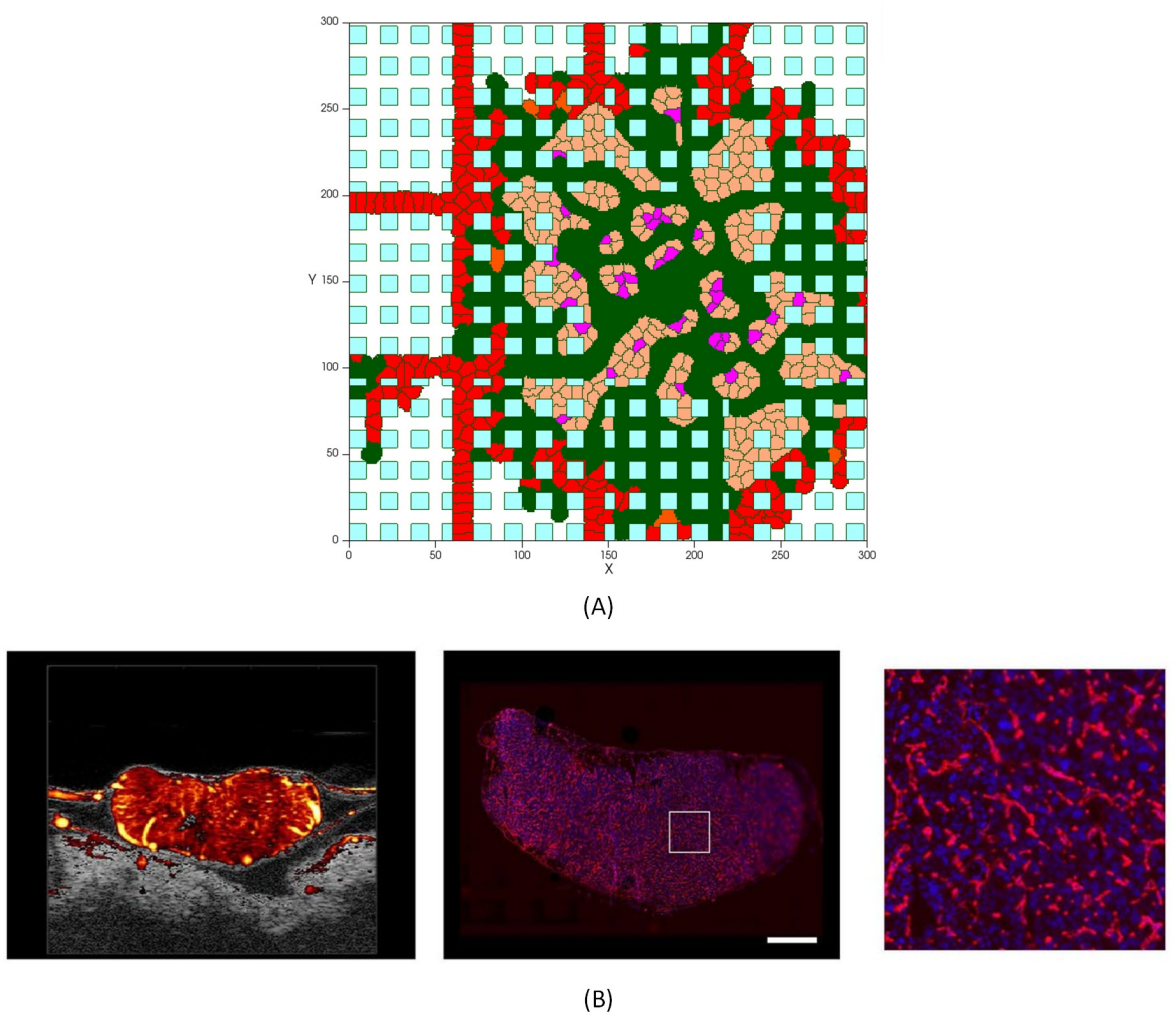
Tumor with a dense capillary network. (A) Simulation results of a high vascularized tumor, with a similar vasculature profile as the experimental image of the network in a tumor [128], (B) Left: Ultrasound Microvessel Imaging; center: fluorescent histology of vessel area around viable tumor cells (scale bar is 1mm); right: enlargement of square area from previous image [128].

It should be noticed that the intratumoral vascularization density (IVD) is investigated as a parameter of malignancy, which is related to aggressive tumor behavior [125–127]. Presence of arterioles both at the border of the lesion and through the core of the tumor has been reported in several experimental studies [128–131]. Fig 14 compares the model result with a tumor microvasculature image, acquired through high-frequency ultrasound microvessel imaging by Huang et al., which was applied to a chicken embryo tumor model of renal cell carcinoma [128].

**Fig 15:**
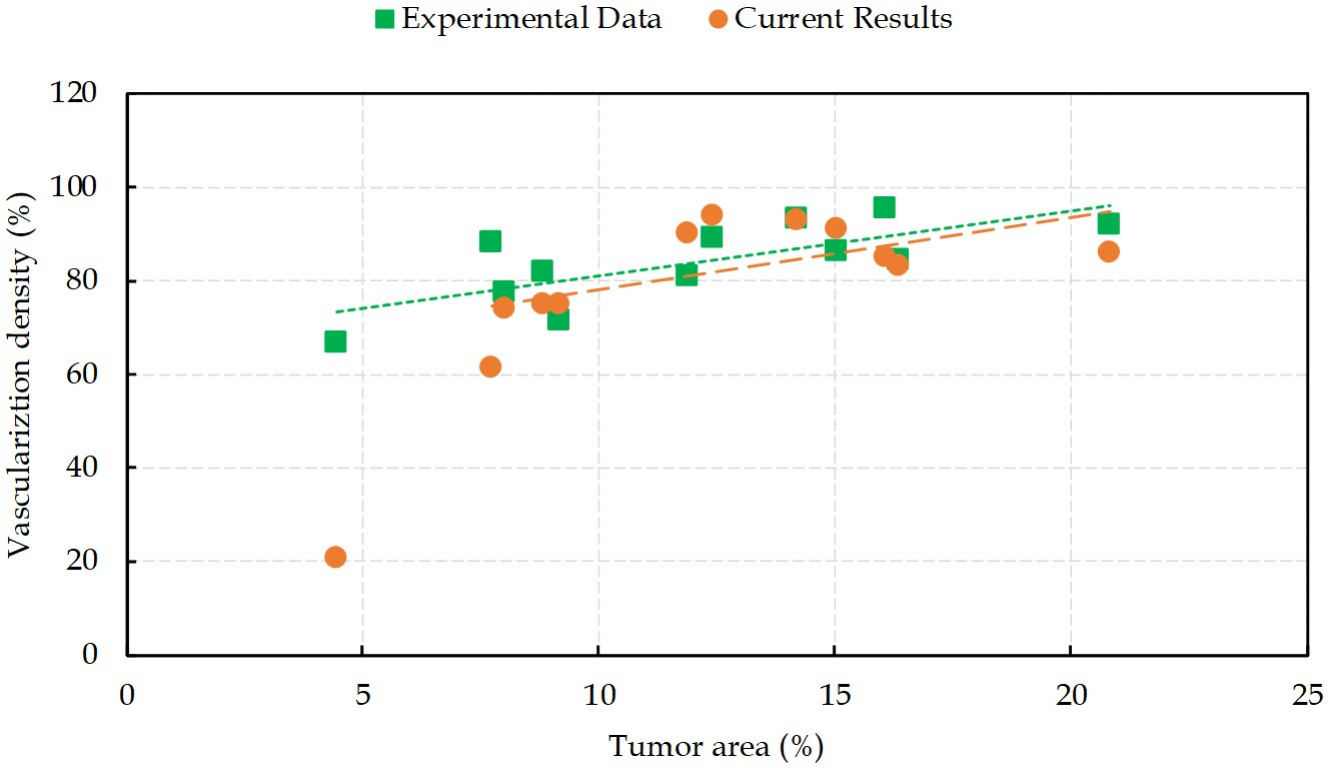
Intratumoral vascularization density in the tumor area. Comparison between simulation results from the present work with the experimental data reported in [128].

When tumor cells gain access to the vessels, they are capable of vascular invasion and to metastasize. The greater the number of tumor induced vessels, the higher the opportunity for cells to enter the blood circulation. This feature is a measure of prognostic in many types of cancers [132]. Hence, to quantify angiogenesis, intratumoral vascularization density is defined as the area of the vessel divided by the total area of the tumor. Fig 15 presents different degrees of vascularization densities in comparison with the reported experimental data in [128]. The results of the model used in this study are in good agreement with IVD experimental measurements. The results correlate a high degree of intratumoral vascularization with a high grade of malignancy, which is confirmed by fluorescent histology in reported data [128].

### Sensitivity analysis

**Fig 16:**
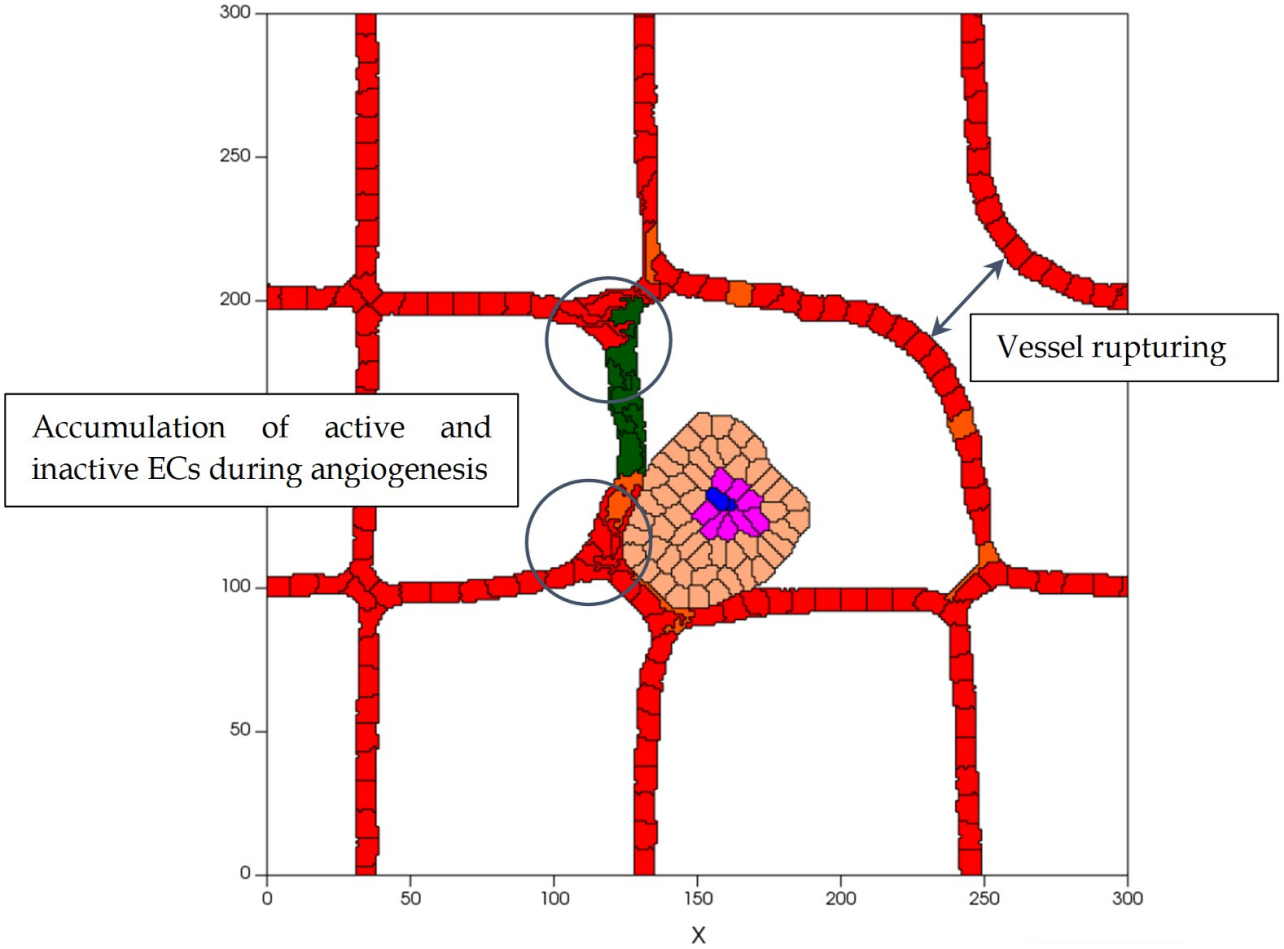
Structure of vascular network for low values of *J*_EC-EC_, on day 7, which leads to an accumulation of active and inactive ECs during angiogenesis and to vessel rupture.

To determine the sensitivity of our findings to the parameters values used, each parameter value is varied, within reasonable ranges, and the changes on the results tracked. Considering various values for the adhesion energy between cells, i.e. *J*, tests were run to study the differences in the process dynamics due to interactions between tumor cells, ECs and their microenvironment. Decreasing the *J* value during tumor development leads to a higher bond between cells, and, consequently, increases the contact area between them. For instance, for *J*_M-M_ ≤ 5 the cells shape is extremely distorted and somewhat unrealistic, and it generates higher tumor cell densities. A similar outcome happens to ECs. *J*_EC-EC_ ≤ 4 causes an accumulation of ECs during sprout development and a rupture in the parent vessels structure (Fig 16). In contrast, increasing *J* is equivalent to weaker cellular adhesion which results in cells reducing their common surface area. For *J*_M-M_ ≥ 15, cells’ shape is, in general, round and there is a separation between cells, as they become farther away from each other as a result of diminished adhesive bonds. Hence, the tumor is formed by a less cohesive population of cells. Moreover, for *J*_EC-EC_ ≥ 15, cellular adhesion forces are too weak to compete with chemotactic forces. Consequently, the tip EC breaks away from the parent vessel.

To ascertain how cell-matrix binding energies affect the tumor evolution process, *J*_m-cell_ variations are considered. For low values of *J*_m-cell_ (≤ 5), there is a significant increase in cell-matrix adhesion force that leads to a relatively strong contact area between the cell membrane and the matrix (both tumor cells and ECs). Lower values of *J*_m-M_ cells lead to an unrealistically distorted shape while at *J*_m-EC_ = 8, the sprout morphology not only is twisted but also the vessels are clearly thicker.

When *J*_m-cell_ increases noticeably, for instance for *J*_m-cell_ = 30, a decreased level of contact guidance is observed. Accordingly, cells are not able to adhere to the matrix and migrate even in the presence of chemotactic stimuli. This can be described as cell-matrix adhesion inhibition. Values within the range 12 ≤ *J*_m-cell_ ≤ 14 build a proper morphology by balancing contact guidance and cell-matrix adhesion energy, which results in insensitive speeds of cells extension and independence to parameter variability.

To assess the compressibility properties, we changed the γ_e_ parameter for different type of cells from 0.3 to 30. Decreasing γ_e_ leads to deviation from the target volume, even changing from the equilibrium condition. On the other hand, large values of γ_e_ cause cell’s resistance to deformation. However, at γ_e_ = 30, the tip EC detaches from the main body of the vessel and migrates toward the tumor. This is because of greater chemotactic force exerted by the tumor than the relative pressure at a tip EC that drives it to reach its target volume. Therefore, values of γ_e_ within ranges 8 ≤ γ_e_ ≤ 13 make no significant change in tumor development.

Moreover, it has been observed that the results are insensitive to the cell-membrane fluctuations when maximum value of *T*_m_ is changed by one order of magnitude. Increasing *T*_m_ causes abnormal changes in cells’ shape that finally leads to cell rupture.

To investigate the sensitivity of the obtained results to the signaling thresholds variability, comparisons between numerical simulation and experimental data were performed. Decreasing the concentration of nutrients and VEGF can be interpreted as high potency of signaling from RTK receptor, similar to the lowering of the integrin threshold, that increases the contribution of integrin signaling pathway. The T_ITG_ represents the integrin binding in which, at the lower values of T_ITG_, fewer bound receptors are sufficient for activation of integrin signaling pathways. Different values of T_ITG_, at its low ranges (<0.2), result in small tumor sizes since more cells undergo apoptosis than surviving. The same event happens for ECs, which prevents sprout development. However, there is an increase in cell survival when the value of T_ITG_ is in the range 0.2 ≤ T_ITG_ ≤ 0.3, thereby, tumor keeps growing and new vessels form around the tumor. The results demonstrate that higher levels of T_ITG_ (>0.35) disrupt not only tumor growth but also affects angiogenesis by interruption of ECs proliferation and migration towards the tumor. This is what also happens during targeted therapy.

Interestingly, the cadherin and Wnt receptor activation thresholds, *T*_Cadherin_ and *T*_Wnt_, regulate, in particular, tumor progression and cells contact inhibition of proliferation, while the activity of Wnt signaling pathway is dependent on cadherin. Cell-cell contact plays a key role at low values of *T*_Cadherin_ so that for *T*_Cadherin_ ≤ 0.2 proliferation is entirely inhibited. The growth of tumor is suppressed and the formation of new vessels is restricted. By increasing the threshold, cells proliferate and the tumor size increases over time. Similarly, while the signaling from VEGF is activated, by increasing *T*_Cadherin_ up to 0.3, the number of proliferating ECs increases and the tumor enters into the vascular growth phase. For *T*_Cadherin_ > 0.3 no significant increase in proliferating cells is observed. The response of tumor to Wnt signaling activity is similar to the activation of the cadherin receptor signaling on account of the communication between Wnt/ß-catenin and cellular adhesion. Our results show that *T*_Wnt_ at 0.15 and higher have a role in cell migration, in which a reduction of cell adhesion increases cell migration. This reduction does not have a considerable effect on cells, and it is consistent with experimental results [133,134].

Moreover, our results show that a VEGF threshold at lower values of ≤ 0.0015 has a decisive role in angiogenesis, since higher values leads to suppression of new vessels growth. High *T*_v_ means that the received signal is not enough to activate the ECs. High activation threshold is likely to deactivate ECs and make them dormant over time. For high thresholds, the average extension speed of new sprouts in a period of 10 hours is measured to be ∼3.65 μm/h, from 5 independent simulations, while the experimental reports estimate this average velocity to be ∼14 μm/h. Similarly, T_RTK_ ≤ 0.005 activates the RTK receptor and its downstream signals in tumor cells and guarantees their survival. However, for TR ≥ 0.005, cells do not receive adequate signals to survive and undergo apoptosis, which result in halted tumor progression.

### Discussing activity thresholds

Analyzing sensitivity of the results to the signal transduction thresholds variations reveals that a higher activation threshold of any receptor means that the initiation of the signaling pathway corresponding to that receptor is improbable. The signal transduction regulation is proposed as possible personalized therapeutic interventions in the treatment of cancer patients. Growth factor receptor activation at *T*_v_ ≤ 0.0015 and *T*_RTK_ ≤ 0.005 regulate cell survival, while increasing thresholds suppress tumor progression. This change is a premise of targeted therapies included in anti-angiogenesis treatment. Moreover, the threshold for cadherin activity, *T*_Cadherin_, can be interpreted as a parameter that controls contact inhibition. For *T*_Cadherin_ < 0.2, the proliferating of cells is completely inhibited, while for *T*_Cadherin_ ≥ 0.2 not only the tumor keeps growing but also the vessels extension velocity increases and reaches a stable value that is insensitive to the threshold. This finding supports the role of VE-cadherin as a regulator of contact inhibition of ECs proliferation, which controls cell-cell adhesion in formation of new vasculature, and E-cadherin role in keeping tumor cells together by facilitating their interaction.

**Fig 17:**
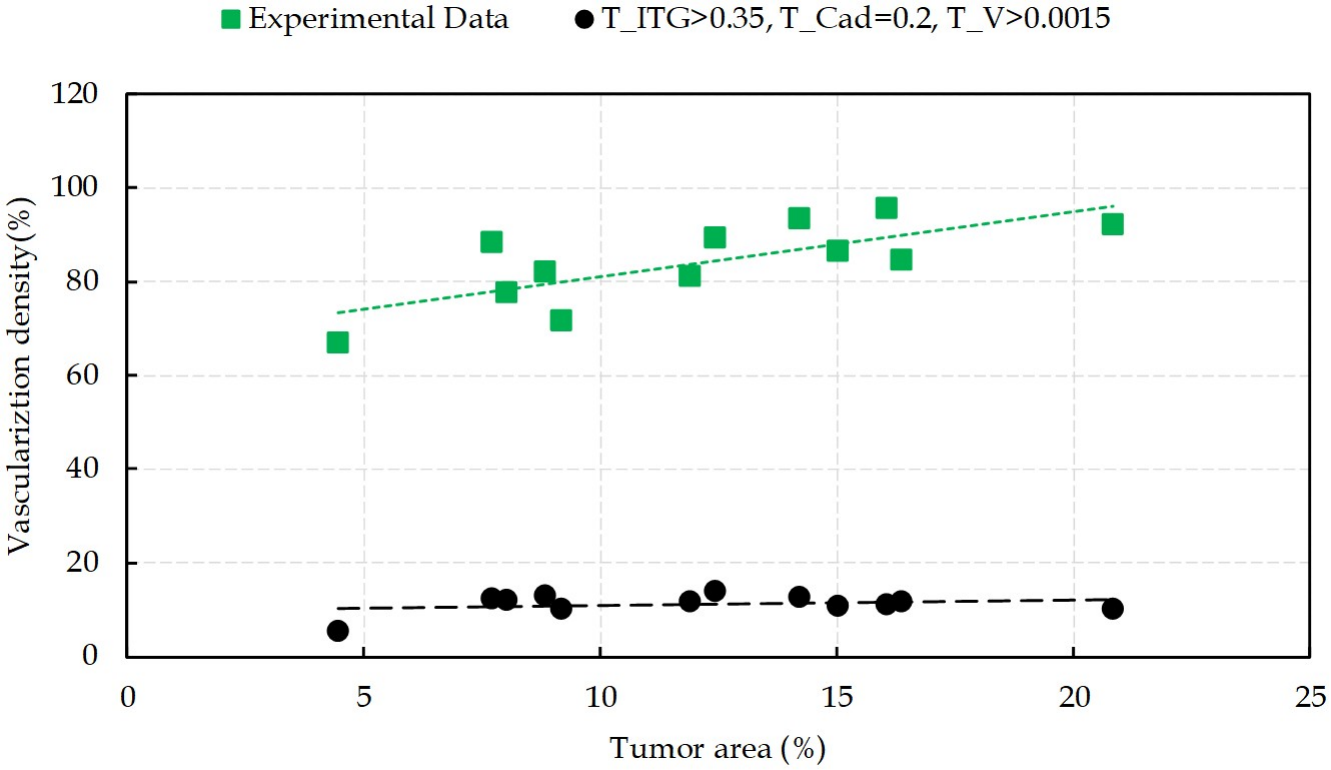
Intratumoral vascularization density as a function of the tumor area, resulting from interventions in signals activation. Comparison between simulation results from present work with experimental data reported in [128].

By choosing the threshold for signaling from integrin receptor, i.e. *T*_ITG_, we find that integrin regulates cells migration when 0.2 ≤ *T*_ITG_ ≤ 0.3. Thereby, it satisfies cell-ECM connection to help vessels migrate toward the tumor at a velocity in accord with the experimental values presented in Fig 4. It should be noted that the migrating cells behavior is combined with proliferation, which means that migrating cells are able to proliferate. Thereby, these aggressive tumor cells move toward the vessels, and angiogenesis occurs based on the two mechanisms of ECs proliferation and migration. Increasing the threshold (T_ITG_ ≥ 0.35), delays the activation of integrin receptor, which results in cells apoptosis and angiogenesis ceases, as reported in several experimental studies [107–110,135,136]. Hence, intervention in the integrin receptor activity, and thereby in its downstream signaling pathways, interrupts cell binding to the ECM and suppresses cell migration by inducing apoptosis. Focusing on the pharmacological role of each receptor in cancer treatment, we developed our model to investigate targeted therapy. Fig 17 shows the effect of changes in the activation of signals in the intratumoral vascularization density.

### Blocking signaling pathways

**Fig 18:**
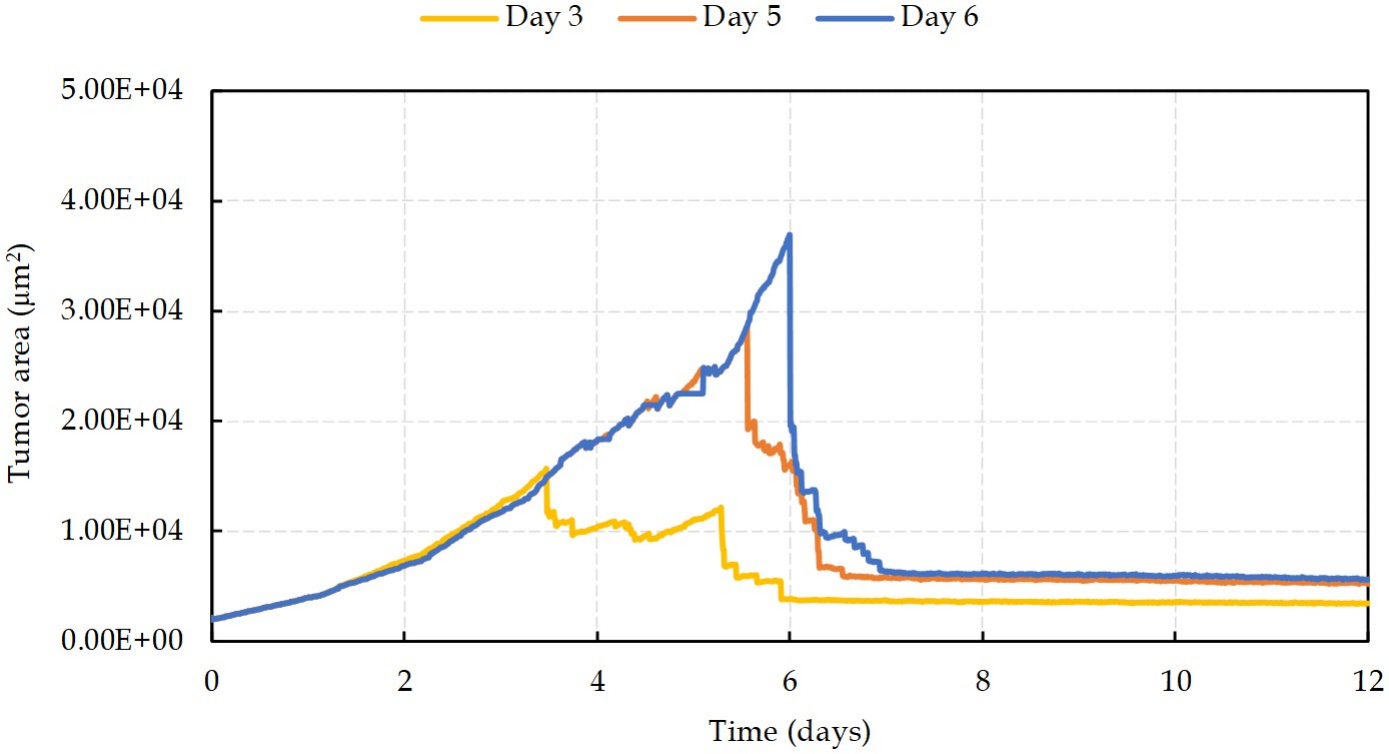
Tumor area evolution with therapy for various treatment initiation days. In all three cases there is a significant initial area decrease on the first day of therapy.

Signaling pathways that suppress tumor growth are good candidates for potential therapeutic targets [79,137,138]. Redundancy of cancer signaling pathways forces the development of a new generation of therapies and anticancer drugs that are based on inhibiting and blocking several signaling mechanisms. More recently, the role of key signaling pathways in various types of cancers was investigated to better understand the mechanisms of specific inhibitors that target those pathways. Inhibition of the pathways Ras/Raf/MEK/ERK and PI3K/PTEN/Akt/mTOR, that are frequently activated because of aberrant regulation of upstream growth factor receptors (RTKs), are considered as novel therapeutic approaches [69,113]. This provides opportunities for development of anti-RTK chemotherapeutic drugs, such as Tyrphostins (Tyrosine-kinase inhibitors TKI) like imatinib and gefitinib, or monoclonal antibodies targeting the extracellular domain of RTKs’ subfamilies, such as Trastuzumab [108,109,139]. Integrin, that mediates cell-matrix interactions, is a critical factor for cell survival, whose inhibition is known as a therapeutic agent, is in clinical trials for cancer treatment [140]. Volociximab is a chimeric monoclonal antibody, that has been developed as an integrin-targeted drug [141]. Therefore, targeting integrin induces cells’ apoptosis, as it is reported in our results at the intracellular scale. Similarly, Wnt contribution to cancer progression shows its important pharmacological potential. Targeting the Wnt pathway by a number of therapeutic agents, as Wnt modulators, has been considered in clinical trials. Furthermore, activity of Wnt/ß-Catenin pathway is dependent on cadherin and the communication between ß-catenin and the cell adhesion molecule, cadherin [142]. We incorporate the results of blocking signals from the intracellular scale to cellular scale to simulate the targeted therapy. Hence, the tumor grows and cells keep proliferating, while the receptors activate downstream signals, until the signaling is disrupted on days 3, 5 and 6 (Fig 18). Referring to Fig 3 for the signals blocking code (cases in which receptors are deactivated: 101, 011, 001, 100, 010, 000), abnormal proliferative state of cells is changed and apoptosis is induced. Moreover, results demonstrate that not only cell migration into the tissue is inhibited but also the release of VEGF, and consequent activation of ECs, is stopped. Thus, the tumor area decreases markedly as the signals get blocked, as shown in Fig 18. According to the day of treatment initiation, in all three cases a significant area decrease is noted on the first day of therapy, which is ~43%, ~77% and ~80%, on average, for days 3, 5, and 6, respectively, as compared with the baseline (no therapy). After treatment initiation the area decreases gradually. Finally, the mean tumor area at day 7 is 5536 μm^2^, with a median area decrease of 80%.

**Fig 19:**
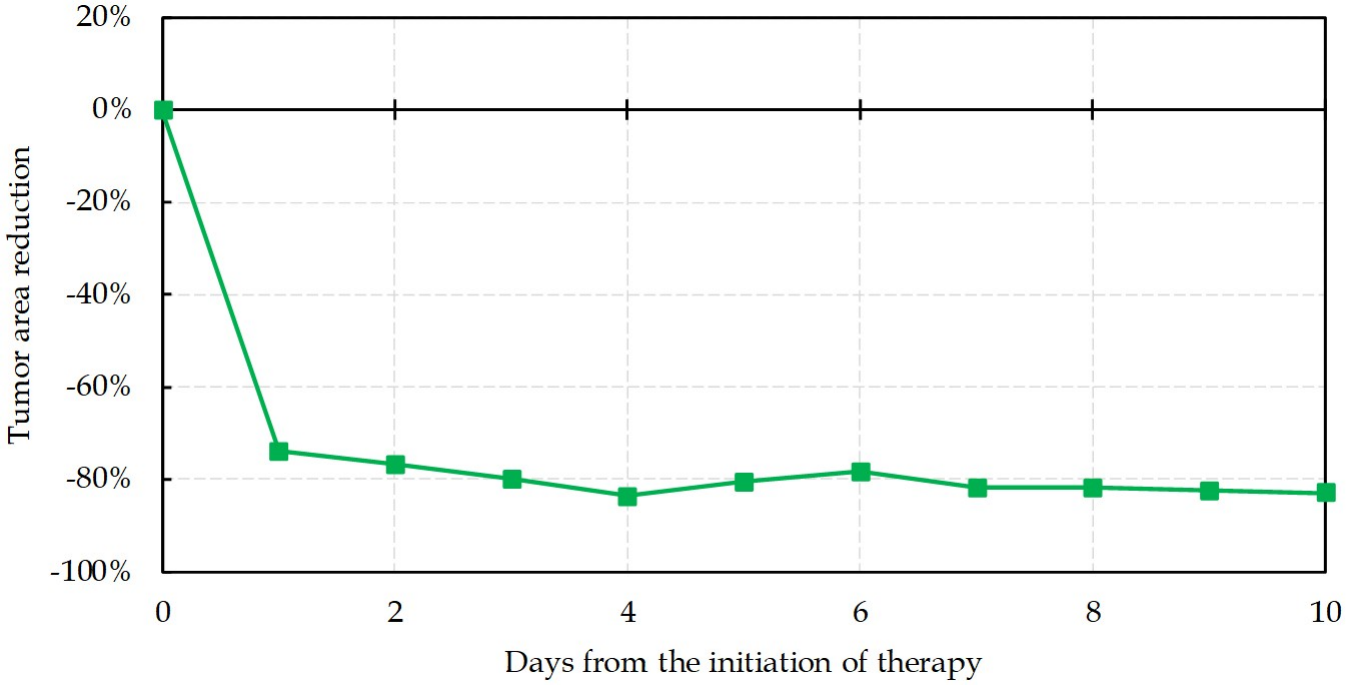
Tumor area percent reduction during 10 days of treatment in comparison with the 25000 μm^2^ baseline area. Blocking the pathways kills cancer cells and prevents tumor growth.

Fig 19 shows the tumor area percent reduction with time with respect to the median baseline tumor area without therapy and a function of the time after therapy initiation. The median baseline is ~25000 μm^2^ and the area changes by 82% on average (range: 78% to 83%) after 10 days of therapy. Again, the initial area decreases significantly and reaches -73% of the baseline after one day. Subsequently, the tumor area decreases gradually, as the signals are blocked and cells undergo apoptosis. Tumor reached the minimum area at day 4 with ~84% decrease compared to baseline.

The numerical data used in all figures are included in S1 Data.

## Conclusion

Tumor growth involves multicellular dynamics at different spatio-temporal scales, including intracellular, intercellular and extracellular processes. The aim of this study is to connect models of avascular and vascular tumor growth in a simulation of carcinogenesis, to mimic experimental assays and, after this first validation, to propose targeted therapy strategies. By including the relevant mechanical and biological properties of endothelial and tumor cells, the model simulates tumor growth and the creation of new capillary vessels, in a simplified but realistic way. This multiscale model also includes a binary network, designed to describe some of the most relevant cellular processes inside each cell regarding tumor evolution, in particular the pathways involved in cell motility, proliferation and death. From this approach it is possible to search for possible inhibitors that can disrupt tumor progress. This makes this model a significant and novel contribution, and opens up a new area of investigation relevant to tumor-targeted treatment strategies, besides simulating the (known) most significant complex biochemical and mechanical interactions between cells and the host microenvironment.

The present study models process at intracellular scale by considering the signaling response to the activation of cell surface receptors and cell adhesion molecules. Once the cell phenotypic behavior, such as proliferation, migration and apoptosis, is determined, cell–cell and cell-matrix interactions are modeled at the intercellular level. Tumor cells access nutrients diffused from nearby vessels, and secrete angiogenic factors to stimulate the growth of new capillaries. This happens at the extracellular scale, with concentrations of nutrients available for cell survival and vascular growth factors present in the domain described by diffusion-reaction PDEs.

In the present study the growth of the avascular and vascular tumor stages are investigated. To capture and describe the biology and physics of the phenomena, normal healthy tissue is considered inside the domain. Comparing each condition, results show that when tumor stimulates ECs activity, and consequently induces angiogenesis, the rate of cancer cells proliferation gets significantly higher, as the sprout forms a closed loop around the tumor (anastomoses). This leads to a substantial increase in the number of cells in each day of progression. Investigating the intratumoral vascularization density (IVD) as a prognostic parameter, the model predicts a malignant tumor growth, in agreement with the experimental observations.

At each time step of the proposed model, cells receive environmental signals and from them gather information on the nutrients availability, the concentration of VEGF and the contact with neighbor cells. These external signals are assessed by each cell via signal transduction and finally determine the cell phenotype. The cells’ behavior, as directed by the signaling pathways, enables us to investigate possible novel therapeutic procedures. These include a combination of therapies targeting specific pathways, which is a significant challenge in cancer treatment. The inhibition of specific molecular targets aims to directly kill cancer cells without affecting normal ones. Although there are major challenges in developing combination therapies using targeted anticancer agents, encouraging advancements show their enormous potential. In this study blocking signals are incorporated in the model to simulate a targeted therapy. The tumor evolution is constantly controlled and determined by the output from the intracellular scale, that is regulated by the intracellular thresholds of the different pathways. These parameters characterize the signaling state of the different receptors at the chosen threshold levels, and impose the cell’s behavior according to the computed output map. The targeted signal blocking prevents excessive cells’ proliferation and is able to drive cells’ apoptosis. Hence, the intracellular parameters that capture cell’s apoptosis are selected to model the treatment. These parameters are never in the range that induce system instability or the rupturing of vessels. Analyzing sensitivity of the results to parameter variations reveals that a higher activation threshold of any receptor means that the initiation of the signaling pathway corresponding to that receptor is improbable. Any disruption in signaling transduction relevant to cells uncontrollable proliferation leads to a significant decrease in tumor area. Results demonstrate that not only cell migration is inhibited but also the release of VEGF, and consequent activation of ECs, is stopped. The tumor median area decreases drastically in comparison with the baseline, within 10 days after blocking receptors activity in the signaling network as a targeted therapy.

## Supporting information

S1 Data Excel spreadsheet containing, in separate sheets, the underlying numerical data for Figs 4, 5, 9, 10, 11, 13, 15, 17, 18 and 19. (XLSX)
